# Global Stabilization of Boolean Networks to Control the Heterogeneity of Cellular Responses

**DOI:** 10.3389/fphys.2018.00774

**Published:** 2018-07-17

**Authors:** Jung-Min Yang, Chun-Kyung Lee, Kwang-Hyun Cho

**Affiliations:** ^1^School of Electronics Engineering, Kyungpook National University, Daegu, South Korea; ^2^Laboratory for Systems Biology and Bio-inspired Engineering, Department of Bio and Brain Engineering, Korea Advanced Institute of Science and Technology, Daejeon, South Korea

**Keywords:** Boolean networks (BNs), global stabilization, sequential control, heterogeneity, systems biology

## Abstract

Boolean networks (BNs) have been widely used as a useful model for molecular regulatory networks in systems biology. In the state space of BNs, attractors represent particular cell phenotypes. For targeted therapy of cancer, there is a pressing need to control the heterogeneity of cellular responses to the targeted drug by reducing the number of attractors associated with the ill phenotypes of cancer cells. Here, we present a novel control scheme for global stabilization of BNs to a unique fixed point. Using a sufficient condition of global stabilization with respect to the adjacency matrix, we can determine a set of constant controls so that the controlled BN is steered toward an unspecified fixed point which can then be further transformed to a desired attractor by subsequent control. Our method is efficient in that it has polynomial complexity with respect to the number of state variables, while having exponential complexity with respect to in-degree of BNs. To demonstrate the applicability of the proposed control scheme, we conduct simulation studies using a regulation influence network describing the metastatic process of cells and the Mitogen-activated protein kinase (MAPK) signaling network that is crucial in cancer cell fate determination.

## 1. Introduction

As a biology-based interdisciplinary field, systems biology is receiving a great interest in recent years as it can investigate complex interactions within biological systems using holistic approaches to biological research (Park et al., 2006; Kim et al., 2007; Murray et al., 2010). Since first proposed by Kauffman (1969), Boolean networks (BNs) have been successfully applied to modeling gene regulatory networks in systems biology. The main reason for utilizing BNs is that they can formulate simplified dynamics of biological networks while capturing the essential characteristics of the networks. Since each gene in the network can be considered to have approximately two levels of activity—active (logical one) or inactive (logical zero), one can define the corresponding Boolean state variables and Boolean logics that serve as state transition functions.

Attractors are the most important factor of BNs as they represent key cellular phenotypes. It is known that finding singleton attractors, or *fixed points* as they are often called, is an NP-hard problem (Akutsu et al., 1998). Nevertheless, many studies exist in the literature on detecting and analyzing attractors in the framework of BNs; see, e.g., Helikar and Rogers (2009); Cheng et al. (2011a); Gonzalez et al. (2006); Zheng et al. (2013); Cheng et al. (2017); Zheng et al. (2016) and references therein.

On the other hand, controlling a cellular behavior is becoming an important issue in systems biology (Liu et al., 2011; Cornelius et al., 2013; Wang et al., 2016). In particular, inducing homogeneous cellular responses is critical to deal with tumor heterogeneity in most of the anti-cancer therapies (Burrell et al., 2013; Mroz et al., 2015; McGranahan and Swanton, 2017). Recent studies confirm that non-genetic heterogeneity is the key driving force for the evolution of cancerous cells (Brock et al., 2009; Shaffer et al., 2017; Dagogo-Jack and Shaw, 2018). In terms of attractors, this is a problem of controlling BNs so that the controlled BN can always converge to one or a smaller number of attractors among all possible ones. It is most desirable if we can reduce the number of undesired attractors selectively. If not, as the second best policy, we can consider the two-step strategy where we first drive the BN toward a global attractor and then transform it into a desired one in the second step. In this way, the desired attractor landscape can have one fixed point.

In this paper, we address the aforementioned problem, termed global stabilization of BNs. The main objective is to determine a set of constant controls that drive the BN toward a unique fixed point. There are many recent results on global stabilization of BNs. Notable among them is Cheng et al. (2011b) that presents necessary and sufficient conditions for global stability of BNs based on a matrix operation called semi-tensor product (STP). In Kim et al. (2013), on the other hand, a minimal set of state variables that make the BN reach a desired attractor is defined as the control kernel, and a general algorithm for the identification of the control kernel is presented. In Zañudo and Albert (2015), attractors are represented by stable motifs and a method is proposed to identify control targets that ensure the convergence of the BN to a desired attractor. The approach in Zañudo and Albert (2015) is remarkable since it combines the structural and functional information of the BN in finding control targets. In Zañudo et al. (2017), a scheme of feedback vertex set control is proposed that drives biological systems described by general non-linear dynamics (including BNs) toward a desired attractor. Recently, Biane and Delaplace (2017) proposed an elegant theoretical scheme that can stabilize a BN in which abduction-based inference is employed to determine constant control inputs using integer linear programming (ILP). While their method guarantees global stabilization, it needs exponential complexity in deriving control targets. Further, if the dynamics of the BN alters by mutations, ILP must be re-formulated. On the other hand, as will be shown later, our method can be applied to BNs having mutations that cause constitutive activity or inactivity of proteins without any modification from the problem setting of a normal case.

In the present study, we adopt the result of Robert (1986) and Cheng et al. (2011b) to determine constant controls that ensure global stability of BNs. In particular, we utilize the sufficient condition that if the influence graph of a BN is acyclic, there is only one fixed point and from each state there should be a trajectory to it. Our method takes a general BN and will search for a set of control inputs so that the resultant influence graph becomes acyclic. Also, the selection of control inputs relies on the canalization effect of a state variable. A canalized state transition function is fixed to a constant when one state variable belonging to the function as an argument is fixed (Kauffman et al., 2004). As the number of state transition functions canalized by a chosen control input increases, the tendency of the controlled BN directing toward global stabilization is becoming higher. In this regard, we will use the canalization effect as another criterion for selecting control inputs.

Note that “global stabilization” in this paper does not mean that the controlled BN converges to a unique fixed point for all the possible combinations of external inputs and mutation profiles. Since activation and inhibition of some genes is determined only by external inputs representing extra-cellular micro-environments or mutations occurring to the genes, the global attractor cannot be always the same. Rather, the essence of the proposed methodology is the ability to provide a consistent set of control inputs that can achieve global stabilization for any given combination of external inputs and mutation profiles. Though the global attractor may vary depending on external inputs and mutations, our solution guarantees global stabilization despite the difference.

The rest of this paper is organized as follows. Basic notations and terminologies of BNs and relevant notions are introduced in section 2. In section 3, we propose an algorithm for determining a set of constant control inputs that make the BN converge to a unique fixed point. Permutation of the adjacency matrix and canalization by state variables are incorporated into an efficient procedure of determining control inputs. To demonstrate the applicability of the proposed control scheme, numerical experiments are conducted in section 4 where we apply the proposed method to a regulation influence network describing the metastatic process of cells (Cohen et al., 2015) and an MAPK signaling network regulating cancer cell fate determination (Grieco et al., 2013). A comparative study with feedback vertex set control, the control kernel method, and the stable motif control is also provided to highlight the efficiency of the proposed scheme.

## 2. Preliminaries

ℕ is the set of natural numbers and [*n*]={1, …, *n*} for *n* ∈ ℕ. For a finite set *A*, |*A*| ∈ ℕ denotes the cardinality of *A*.

A BN with *n* binary state variables *x*_1_, …, *x*_*n*_ ∈ {0, 1} is represented by a Boolean mapping $F={\left(f_{1},\ldots ,f_{n}\right)}^{T}:{\left\{0,1\right\}}^{n}\to {\left\{0,1\right\}}^{n}$ where $f_{i}:{\left\{0,1\right\}}^{n}\to \left\{0,1\right\}$ is the state transition equation of *x*_*i*_. Index *i* and *x*_*i*_ will be used interchangeably for the *i*th state variable. Letting $x={\left(x_{1},\ldots ,x_{n}\right)}^{T}$, we express the state evolution with *x*: = *F*(*x*). Although our study focuses on BNs with synchronous updating, it can be also applied to asynchronously updating BNs.

The connectivity of *F* is described by a Boolean matrix with respect to the influence graph of *F* (Paulevé and Richard, 2012), a topological representation of *F* in which state variables serve as nodes and there is an edge *x*_*i*_ → *x*_*j*_ when *f*_*j*_ depends on *x*_*i*_.

Definition 1. *Given F, the adjacency matrix A*(*F*) *is an n* × *n Boolean matrix whose* (*i*,*j*) *entry A_i,j_*(*F*) *is 1 if there exists a state* ${\left(x_{1},\ldots ,x_{j-1},0,x_{j+1},\ldots ,x_{n}\right)}^{T}$ *such that f_i_*(*x*_1_, …, *x*_*j*−1_, 0, *x_j_* + 1, …, *x_n_*) ≠ *f_i_*(*x*_1_, …, *x*_*j*−1_, 1, *x*_*j* + 1_, …, *x_n_*)*; otherwise, A_i,j_*(*F*) = 0.

*A*_*i,j*_(*F*) is equal to 1 when *x*_*i*_ is directly affected by *x*_*j*_. Letting *A*(*F*) = (*a*_*i,j*_), denote by $a_{i}^{r}\in {\left\{0,1\right\}}^{1\times n}$ and $a_{j}^{c}\in {\left\{0,1\right\}}^{n\times 1}$ the *i*th row vector and *j*th column vector of (*a*_*i,j*_), respectively. The norm of each vector is defined as:

$$\begin{array}{l} |a_{i}^{r}|=\overset{n}{\underset{j\;=\;1}{\sum }}a_{i,j}\\ |a_{j}^{c}|=\overset{n}{\underset{i\;=\;1}{\sum }}a_{i,j} \end{array}$$

$\left|a_{i}^{r}\right|$ and $\left|a_{j}^{c}\right|$ are equal to the number of all the incoming and outgoing edges of *x*_*i*_ and *x*_*j*_, respectively. For the row vector $a_{i}^{r}$ and the column vector $a_{j}^{c}$, we define additional parameters:

$$\begin{array}{l} d\left(a_{i}^{r}\right)=\overset{i}{\underset{j\;=\;1}{\sum }}a_{i,j}\\ d\left(a_{j}^{c}\right)=\overset{j}{\underset{i\;=\;1}{\sum }}a_{i,j} \end{array}$$

to denote the sum of all one entries from the first to *i*th position of $a_{i}^{r}$ and from the first to *j*th position of $a_{j}^{c}$, respectively.

Example 1. *Consider the following synthetic BN* $F={\left(f_{1},\ldots ,f_{8}\right)}^{T}$ *with*

(1) $$\begin{array}{l} f_{1}\left(x\right)=\neg x_{3}\wedge x_{7}\wedge \neg x_{8}\\ f_{2}\left(x\right)=\left(x_{5}\vee x_{6}\right)\wedge \neg x_{8}\\ f_{3}\left(x\right)=x_{8}\\ f_{4}\left(x\right)=x_{2}\wedge \neg x_{7}\\ f_{5}\left(x\right)=x_{2}\vee x_{4}\\ f_{6}\left(x\right)=x_{3}\wedge \neg x_{8}\\ f_{7}\left(x\right)=x_{2}\wedge \neg x_{8}\\ f_{8}\left(x\right)=\neg \left(x_{1}\vee x_{2}\right)\wedge \left(x_{4}\vee x_{7}\right) \end{array}$$

*where* ¬, ∧*, and* ∨ *are negation, conjunction, and disjunction operation, respectively. In view of Definition 1, the adjacency matrix* $A\left(F\right)=\left(a_{i,j}\right)\in {\left\{0,1\right\}}^{8\times 8}$ *is derived as*

$$\begin{array}{l} \left(a_{i,j}\right)=\left(\begin{array}{cccccccc} 0 & 0 & 1 & 0 & 0 & 0 & 1 & 1\\ 0 & 0 & 0 & 0 & 1 & 1 & 0 & 1\\ 0 & 0 & 0 & 0 & 0 & 0 & 0 & 1\\ 0 & 1 & 0 & 0 & 0 & 0 & 1 & 0\\ 0 & 1 & 0 & 1 & 0 & 0 & 0 & 0\\ 0 & 0 & 1 & 0 & 0 & 0 & 0 & 1\\ 0 & 1 & 0 & 0 & 0 & 0 & 0 & 1\\ 1 & 1 & 0 & 1 & 0 & 0 & 1 & 0 \end{array}\right) \end{array}$$

*F has three attractors* σ_1_–σ_3_ *where* σ_1_ *and* σ_2_ *are fixed points and* σ_3_ *is a cycle with length 2:*

$$\begin{array}{l} \sigma_{1}=\left(1,1,0,0,1,0,1,0\right)\\ \sigma_{2}=\left(0,0,0,0,0,0,0,0\right)\\ \sigma_{3}=\left(0,1,0,0,1,1,0,1\right)⇆\left(0,0,1,1,1,0,0,0\right) \end{array}$$

**Figure 1:**
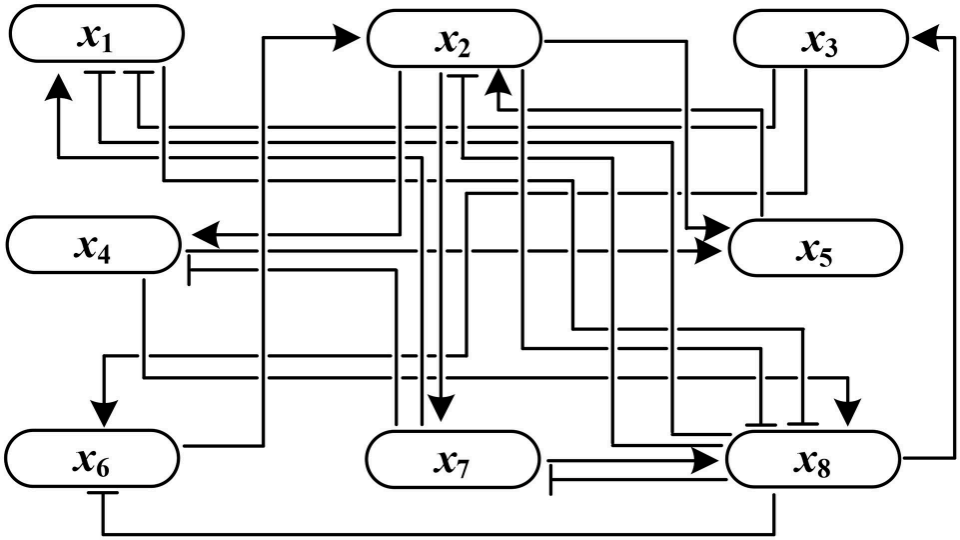
Influence graph of *A*(*F*).

*Figure 1 shows the influence graph of A*(*F*)*, where the arrows with pointed heads represent activation and those with bar heads represent inhibition.*

In Cheng et al. (2011b), the necessary and sufficient condition for global stability of *F* to a fixed point is presented in terms of the adjacency matrix.

Theorem 1. *A BN F globally converges to a unique fixed point if and only if there exists k* ∈ ℕ *such that* $A\left(F^{k}\right)=0_{n\times n}$ *where F^k^ denotes the kth iterate of F.*

$A\left(F^{k}\right)=0_{n\times n}$ implies that all the edges between state variables are disconnected after the *k*th iteration. Hence *F* will reach a unique fixed point for any initial state. However, since the computation of *F*^*k*^ has exponential complexity with respect to *n*, this criterion is difficult to apply when *n* is large. Cheng et al. (2011b) also presents a sufficient condition for the existence of a global attractor with polynomial complexity.

Theorem 2. *For a BN F, assume that there exists k* ∈ ℕ *such that* (*A*(*F*))^*k*^ = **0***, where* (*A*(*F*))^*k*^ *denotes Boolean power in which the sum and product operations in the matrix multiplication are logical OR and AND, respectively. Then, F globally converges to a unique fixed point.*

The existence of *k* ∈ ℕ leading to (*A*(*F*))^*k*^ = **0** can be determined by checking whether *A*(*F*) falls under a specific category as stated below (Robert, 1986).

Theorem 3. *For a BN F, there exists k* ∈ ℕ *such that* (*A*(*F*))^*k*^ = **0** *if and only if a permutation matrix H* ∈ {0, 1}^*n* × *n*^ *exists such that* $H^{T}\times_{B}A\left(F\right)\times_{B}H$ *is a strictly lower triangular (equivalently, upper triangular) matrix, where ‘* × *_B_’ denotes the Boolean product.*

$H^{T}\times_{B}A\left(F\right)\times_{B}H$ signifies that state variables of *F* are reordered according to *H*. If $H^{T}\times_{B}A\left(F\right)\times_{B}H$ is strictly lower triangular, the influence graph of *F* encoded by the adjacency matrix *A*(*F*) turns out to be acyclic as addressed in Robert (1986). Theorem 2 and Theorem 3 stipulate that with this condition, *F* will converge to a unique fixed point after some iterations *k* ∈ ℕ. Further, (*A*(*F*))^*k*^ = **0** is a sufficient condition for global stabilization since *A*(*F*^*k*^) ≤ (*A*(*F*))^*k*^ for all *k*.

Example 2. *Consider a BN* $F={\left(f_{1},f_{2},f_{3},f_{4}\right)}^{T}$ with

$$\begin{array}{l} f_{1}\left(x\right)=x_{2}\vee x_{3}\\ f_{2}\left(x\right)=\neg x_{4}\\ f_{3}\left(x\right)=x_{2}\wedge x_{3}\\ f_{4}\left(x\right)=\neg x_{3} \end{array}$$

*Assume that x*_3_ *is fixed to 1 as a control input. With x*_3_ = 1*, The second and third iterate F*^2^ *and F*^3^ *are derived as*

$$\begin{array}{l} F^{2}=\left\{ \begin{array}{l} x_{1}:=\neg x_{4}\vee 1\;\\ x_{2}:=x_{3}\;\\ x_{3}:=1\;\\ x_{4}:=0\; \end{array}\right.F^{3}=\left\{ \begin{array}{l} x_{1}:=1\;\\ x_{2}:=1\;\\ x_{3}:=1\;\\ x_{4}:=0\; \end{array}\right. \end{array}$$

*Since* $A\left(F^{3}\right)=0_{4\times 4}$*, by Theorem 1 the BN globally stabilizes to (1,1,1,0)^T^ in three steps from any initial state. The adjacency matrix of F with x*_3_ = 1 *is*

$$\begin{array}{l} A\left(F\right)=\left(\begin{array}{cccc} 0 & 1 & 1 & 0\\ 0 & 0 & 0 & 1\\ 0 & 0 & 0 & 0\\ 0 & 0 & 1 & 0 \end{array}\right) \end{array}$$

*It is easy to compute that* ${\left(A\left(F\right)\right)}^{\left(4\right)}=0_{4\times 4}$*. Hence global stability of the BN is confirmed again by Theorem 2. The latter can be proved by employing the following permutation matrix*

$$\begin{array}{l} H=\left(\begin{array}{cccc} 1 & 0 & 0 & 0\\ 0 & 1 & 0 & 0\\ 0 & 0 & 0 & 1\\ 0 & 0 & 1 & 0 \end{array}\right) \end{array}$$

*that switches the order of x*_3_ *and x*_4_*. Since*

$$\begin{array}{l} H^{T}\times_{B}A\left(F\right)\times_{B}H=\left(\begin{array}{cccc} 0 & 1 & 0 & 1\\ 0 & 0 & 1 & 0\\ 0 & 0 & 0 & 1\\ 0 & 0 & 0 & 0 \end{array}\right) \end{array}$$

*is strictly upper triangular, by Theorem 3 k* ∈ ℕ *exists such that* ${\left(A\left(F\right)\right)}^{\left(k\right)}=0_{4\times 4}$ *(k turns out to be 4 in this case).*

For *x* and *P* = {(*i*_1_, *u*_1_), …, (*i*_|*P*|_, *u*_|*P*|_)} ⊂ [*n*] × {0, 1}, let ${\hat{x}}^{P}$ be the state vector in which each *x*_*i*_*k*__ is fixed to a constant *u*_*k*_, *k* = 1, …, |*P*|. If *P* = ∅, ${\hat{x}}^{\emptyset }=x$. For later usage, let *P*_[*n*]_ = {*i*_1_, …, *i*_|*P*|_} ⊂ [*n*]. ${\hat{x}}^{P}$ stands for the state vector wherein some state variables are selected as constant control inputs or are canalized by other control inputs. This notation will be utilized in developing the algorithm for global stabilization.

Assume that in a BN *F*, *x*_*i*_*k*__ has been fixed to *u*_*k*_, *k* = 1, …, |*P*|, as characterized by *P*. Then for *x*_*j*_ and *f*_*i*_ where *i,j* ∈ [*n*] − *P*_[*n*]_ and *i* ≠ *j*, *x*_*j*_ is called a *canalizing variable* of the transition function *f*_*i*_ if there exist *u, v* ∈ {0, 1} such that setting *x*_*j*_ = *u* in ${\hat{x}}^{P}$ canalizes $f_{i}\left({\hat{x}}^{P}\right)$ to *v*. Note that all successive canalizations by *x*_*j*_ = *u* are considered in checking the canalization of *f*_*i*_, namely, more than one transition function may be canalized in a sequential way as the result of setting *x*_*j*_ = *u*. *f*_*i*_ is said to be a (*u, v*)-canalized transition function of *x*_*j*_ with respect to ${\hat{x}}^{P}$ [a similar definition is presented in Cheng et al. (2011a)].

To quantify the canalization effect of a state variable, denote by *C*_*j*_(*P*; *u*) ⊂ [*n*] − *P*_[*n*]_ the index set of all (*u*, ^*^)-canalized transition functions of *x*_*j*_ with respect to ${\hat{x}}^{P}$ that are derived by setting *x*_*j*_ = *u*. For instance, if *x*_*j*_ = *u* canalizes *f*_*j*_ as well as another transition function $f_{j^{\prime }}$, we have $C_{j}\left(P;u\right)=\left\{j,j^{\prime }\right\}$. It is convenient to elucidate which setting among *x*_*j*_ = 0 and *x*_*j*_ = 1 yields greater canalization effect. To this end, define

$$\begin{array}{l} T_{j}\left(P\right)=\max\left(\left|C_{j}\left(P;0\right)|,|C_{j}\left(P;1\right)\right|\right) \end{array}$$

as the *canalization number* of *x*_*j*_ with respect to ${\hat{x}}^{P}$. *T*_*j*_(*P*) equals the maximum number of canalized transition functions of *x*_*j*_ that are found for all state variables in [*n*] − *P*_[*n*]_. But to describe our algorithm of global stabilization, we often need to restrict the state variables of interest to a subset of [*n*] − *P*_[*n*]_. Formally, for *Q* ⊆ [*n*] − *P*_[*n*]_ define *T*_*j*_(*P, Q*), where *j* ∈ *Q*, as

$$\begin{array}{l} T_{j}\left(P,Q\right)=\max\left(\left|C_{j}\left(P;0\right)\cap Q|,|C_{j}\left(P;1\right)\cap Q\right|\right) \end{array}$$

*T*_*j*_(*P, Q*) represents the maximum number of canalized transition functions of *x*_*j*_ that are searched only among state variables of *Q*.

Example 3. *Global stabilization by the control input x*_3_ = 1 *in Example 2 can be interpreted as canalization. In view of Example 2, we can set P* = *and Q* = {1, 2, 3, 4}*. Once x*_3_ *is fixed to 1, x*_4_ *is also fixed to 0 in the second iterate F*^2^. *Further, x*_3_ *and x*_4_ *canalize x*_1_ *and x*_2_ *to 1 in the third iterate F*^3^. *Hence C*_3_(*P*; 1) = 3. *Similarly, C*_3_(*P*; 0) = 3 *and thus T*_3_(*P*, *Q*) = max(3, 3) = 3.

## 3. Methods

### 3.1. Global stabilization

Although the criterion of Theorem 3 is sufficient but not necessary, it can serve as a practical tool to determine control inputs to complex biological networks since *A*(*F*) has a polynomial complexity with respect to *n*. Specifically, to derive *A*(*F*) from the influence graph of *F* with *n* nodes, one must check whether any pair of nodes are adjacent with each other. Hence *A*(*F*) is computed in *O*(*n*^2^). Based on Theorem 3, we now propose a scheme of deriving a set of control inputs that guarantee global stabilization of a BN *F*. Theorem 3 implies that if the adjacency matrix or one of its permuted matrices is strictly lower triangular, *F* converges to a unique fixed point. To utilize this result, we first reorder state variables of *F* so that the permuted adjacency matrix can be as similar to a strictly lower triangular matrix as possible. If the permuted adjacency matrix turns out to be strictly lower triangular, no assignment of control inputs is necessary. Otherwise, we select in a sequential way a set of state variables that will be used as control inputs. In terms of the graph representation, the latter scheme is equal to making the influence graph of *F* acyclic by fixing some nodes, thus removing their input edges and potentially breaking cycles.

Once a state variable *x*_*i*_ is selected as a control input, all the incoming edges of *x*_*i*_ are disconnected, leading to $a_{i}^{r}=0_{1\times n}$. In terms of global stabilization, we can also regard that the outgoing edges of *x*_*i*_ are ‘disconnected’ since the influence of *x*_*i*_ on other state variables becomes constant as does the value of *x*_*i*_. Hence we will set $a_{i}^{c}=0_{n\times 1}$ in our algorithm for determining control inputs. In this regard, it would be best if we first select the state variable that has the greatest outgoing edges in its upper right entries of the (permuted) adjacency matrix. Moreover, we must consider the canalization effect of the selected state variable. If the transition function of another state variable is canalized by the selected state variable, all the corresponding entries of the adjacency matrix also degenerate into zeros. How many state variables are canalized, as is quantified by the canalization number, will be also utilized as a criterion to select the control input. The following algorithm is the main result of this paper.

Algorithm 1. *Derivation of control inputs that make the adjacency matrix strictly lower triangular:*

*Given a BN F with the adjacency matrix A*(*F*) = (*a*_*i*,*j*_)*, we determine a set of control inputs that ensures global stability of F. Set P* = *and Q* = [*n*].

1. *Permute* (*a*_*i*,*j*_) *and update Q as follows.*
  - *Sort the row vectors into an ascending order of the row vector norm. Letting i*(1), …, *i*(*n*) *be the sorted indices, we have*
    $$\begin{array}{l} \left|a_{i\left(1\right)}^{r}|\leq |a_{i\left(2\right)}^{r}|\leq \cdots \leq |a_{i\left(n\right)}^{r}\right| \end{array}$$
  - *Permute* (*a*_*i*,*j*_) *according to i*(1), …, *i*(*n*)*, i.e., reorder the state variables so that x*_*i*(*k*)_ *is placed on the kth position for all k* ∈ [*n*]. *Let* (ã_*i*,*j*_) *be the permuted matrix of* (*a*_*i*,*j*_).
  - *Set* $Q=Q-\left\{j\in Q|d\left({ã}_{j}^{c}\right)=0\right\}$*.*
2. Search for j^*^ ∈ *Q as follows.*
  - *Let K* ⊂ *Q be the set of indices such that*
    $$\begin{array}{l} k=\arg\underset{j\in Q}{\max}d\left({ã}_{j}^{c}\right)\;\forall k\in K \end{array}$$
  - Among the entries of K, find j^*^ *such that*
    $$\begin{array}{l} j^{*}=\arg\underset{k\in K}{\max}T_{k}\left(P,Q\right) \end{array}$$
3. *Modify* (ã_*i*,*j*_) *and update P and Q as follows.*
  - Let u^*^ ∈ {0, 1} *be the value of* $x_{j^{*}}$ *such that* $T_{j^{*}}\left(P,Q\right)=|C_{j^{*}}\left(P;u^{*}\right)\cap Q|$*.*
  - *Set*
    $$\begin{array}{l} {ã}_{j^{*}}^{r}={ã}_{h}^{r}=0_{1\times n}\\ {ã}_{j^{*}}^{c}={ã}_{h}^{c}=0_{n\times 1}\;\forall h\in C_{j^{*}}\left(P;u^{*}\right)\cap Q \end{array}$$
  - *Update P and Q by*
    $$\begin{array}{l} P=P\cup \left\{\left(j^{*},u^{*}\right)\right\}\\ Q=Q-\left\{j^{*}\right\}\cup C_{j^{*}}\left(P;u^{*}\right) \end{array}$$
4. *If* (ã_*i*,*j*_) *is strictly lower triangular, terminate the algorithm. The solution to global stabilization of F is*
  $$\begin{array}{l} x_{j_{1}}=u_{1},\ldots ,x_{j_{\left|P\right|}}=u_{\left|P\right|} \end{array}$$

where *P* = {(*j*_1_, *u*_1_), …, (*j*_|*P*|_, *u*_|*P*|_)}. Otherwise, return to Step 2.

In the above algorithm, *P* denotes the set of selected control inputs so far and *Q* represents eligible candidates that can be selected as control inputs in the next step (*P*_[*n*]_ ∩ *Q* = ∅). Step 1 describes the permutation of (*a*_*i,j*_) by reordering state variables. Since the permuted adjacency matrix must be akin to a strictly lower triangular one, we reorder *x*_*i*_'s in an ascending order of the norm of the corresponding row vectors, that is, those with more incoming edges are placed on later positions. If $d\left({ã}_{j}^{c}\right)=0$, *x*_*j*_ needs not be selected as a control input since the present form of its column vector ${ã}_{j}^{c}$ is already a component of a strictly lower triangular matrix. Thus *x*_*j*_ is removed from the candidate set *Q* (Step 1.c).

In Step 2, we derive the index j^*^ of the state variable that, if selected as a control input, can modify (ã_*i,j*_) so that the changed matrix approaches a strictly lower triangular matrix the most. The best candidate would be the one having the most outgoing edges in its upper right entries, which is represented by $d\left({ã}_{j}^{c}\right)$ (Step 2.a). If more than one state variable have the maximum $d\left({ã}_{j}^{c}\right)$, we choose the variable that has the greatest canalization number (Step 2.b), since the corresponding row and column vectors of all canalized state variables degenerate into zeros (Step 3.b), hence contributing to the modification of (ã_*i,j*_) to a strictly lower triangular matrix.

Once selected as a control input or canalized by any selected control input, the state variable must be excluded from the candidate set *Q* (Step 3.c). If the modified adjacency matrix is strictly lower triangular, the assignment of constant controls in *P* is the solution to global stabilization (Step 4). Otherwise, the foregoing steps are iterated until the solution is derived.

**Figure 2:**
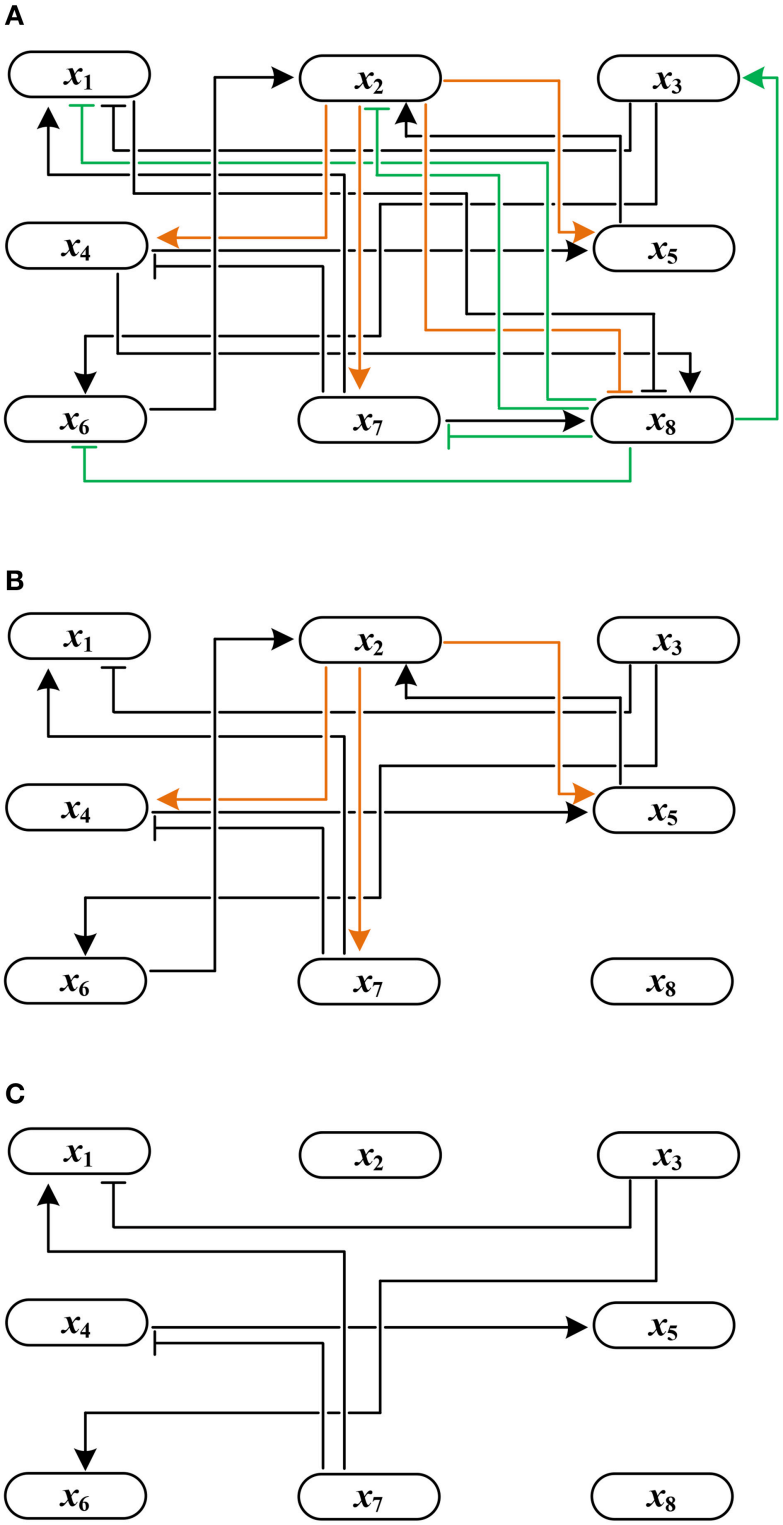
Change of the influence graph of *F*: **(A)** before control, **(B)** removal of incoming/outgoing edges of *x*_8_ by setting *x*_8_ = 1, and **(C)** removal of incoming/outgoing edges of *x*_2_ by canalization (the resultant influence graph becomes acyclic).

Example 4. *Using Algorithm 1, let us derive the solution to global stabilization of F in Example 1. According to Step 1.a–b of Algorithm 1, we first permute* (*a*_*i*,*j*_) *by reordering state variables in an ascending order of the norm of their row vectors. The permuted adjacency matrix* (ã_*i*,*j*_) *is shown in Table 1. Here,* $|a_{8}^{r}|=4$*,* $|a_{2}^{r}|=3$*, and so on. Since* $d\left({ã}_{j}^{c}\right)=0$ *for all j* ∈ {3, 7, 6, 4, 5, 1}*, we set Q* = {2, 8} *by Step 1.c. This means that only the outgoing edges of x*_2_ *and x*_8_ *are in the upper right positions of the permuted* (ã_*i*,*j*_)*. Figure 2A illustrates this topology where the outgoing edges of x*_2_ *and x*_8_ *are drawn in orange and green, respectively. Since* $d\left({ã}_{8}^{c}\right)=5>d\left({ã}_{2}^{c}\right)=3$, j^*^ = 8 *is selected as the first control input by Step 2. As the value of x*_8_ *is made constant, the incoming/outgoing edges of x*_8_ *are removed from the influence graph as shown in Figure 2B. Referring to (1), further, f*_2_ *is* (1, 0)*-canalized by x*_8_*. Hence x*_8_ = 1 *is the value that maximally canalizes the remaining variables of Q—single element x*_2_ *in this case. Applying Step 3.b, we modify* (ã_*i*,*j*_) *to*

$$\begin{array}{l} \left({ã}_{i,j}\right)=\left(\begin{array}{cccccccc} 0 & 0 & 0 & 0 & 0 & 0 & 0 & 0\\ 0 & 0 & 0 & 0 & 0 & 0 & 0 & 0\\ 1 & 0 & 0 & 0 & 0 & 0 & 0 & 0\\ 0 & 1 & 0 & 0 & 0 & 0 & 0 & 0\\ 0 & 0 & 0 & 1 & 0 & 0 & 0 & 0\\ 1 & 1 & 0 & 0 & 0 & 0 & 0 & 0\\ 0 & 0 & 0 & 0 & 0 & 0 & 0 & 0\\ 0 & 0 & 0 & 0 & 0 & 0 & 0 & 0 \end{array}\right) \end{array}$$

**Table 1:** Permuted adjacency matrix (ã_*i,j*_).

**$\left|a_{i}^{r}\right|$**	***x*_3_**	***x*_7_**	***x*_6_**	***x*_4_**	***x*_5_**	***x*_1_**	***x*_2_**	***x*_8_**
1	0	0	0	0	0	0	0	1
2	0	0	0	0	0	0	1	1
2	1	0	0	0	0	0	0	1
2	0	1	0	0	0	0	1	0
2	0	0	0	1	0	0	1	0
3	1	1	0	0	0	0	0	1
3	0	0	1	0	1	0	0	1
4	0	1	0	1	0	1	1	0

*Since the above matrix is strictly lower triangular, we terminate the algorithm by determining the solution x*_8_ = 1*. The resultant influence graph becomes acyclic as shown in Figure 2C. The global fixed point obtained by the control input x*_8_ = 1 is x^*^ = (0, 0, 1, 0, 0, 0, 0, 1)^*T*^*, which differs from* σ_1_–σ_3_ *derived in Example 1.*

To discuss computational complexity of Algorithm 1, let *s* ∈ ℕ be the maximum number of incoming edges of a node in *F*. In Step 1.a, sorting the row vectors needs *n*^2^ operations in the worst case. Since Step 1.b and Step 1.c can be done in one operation, respectively, Step 1 has the maximum *n*^2^ + 2 operations. Step 2.a needs *n* operations in the worst case. In Step 2.b, on the other hand, we need to derive the canalization number of each state variable. For a state variable with *l* incoming edges, we must check whether the corresponding state transition function is fixed to a constant for all 2^*l*−1^ combinations of arguments (one argument is the canalizing variable). Hence Step 2.b needs *n*2^*s*−1^ operations in the worst case. Step 3 has four operations (Step 3.a needs two operations to determine u^*^), and finally Step 4 has just one operation. Combining these factors, we conclude that Algorithm 1 can be computed in *O*(*n*^2^ + *n*2^*s*−1^). In other words, Algorithm 1 has polynomial complexity with respect to the number of state variables, while having exponential complexity with respect to the number of incoming edges. When the considered BN has a state variable with a huge number of incoming edges, applying Algorithm 1 may be computationally demanding. Still, Algorithm 1 is useful since it is known that BNs representing biological systems are very sparse in general—the average degree of a node is about two (Leclerc, 2008).

As duality of making a strictly lower triangular matrix, we can adjust Algorithm 1 so as to search for a set of control inputs that make the resulting adjacency matrix strictly upper triangular. To this end, we reorder state variables according to an ascending order of the column vector norm $\left|a_{j}^{c}\right|$, find the candidate control input that has the greatest $\left|d\left(a_{i}^{r}\right)\right|$, and so on. The following algorithm is analyzed in a similar way to Algorithm 1.

Algorithm 2. *Derivation of control inputs that make the adjacency matrix strictly upper triangular:*

*Given a BN F with the adjacency matrix A*(*F*) = (*a*_*i*,*j*_)*, we determine a set of control inputs that ensures global stability of F. Set P* = *and Q* = [*n*]*.*

1. *Permute* (*a*_*i*,*j*_) *and update Q as follows.*
  - *Sort the column vectors into an ascending order of the column vector norm. Letting j*(1), …, *j*(*n*) *be the sorted indices, we have*
    $$\begin{array}{l} \left|a_{j\left(1\right)}^{c}|\leq |a_{j\left(2\right)}^{c}|\leq \cdots \leq |a_{j\left(n\right)}^{c}\right| \end{array}$$
  - *Permute* (*a*_*i*,*j*_) *according to j*(1), …, *j*(*n*)*, i.e., reorder the state variables so that x*_*j*(*k*)_ *is placed on the kth position for all k* ∈ [*n*]*. Let* (ã_*i*,*j*_) *be the permuted matrix of* (*a*_*i*,*j*_)*.*
  - *Set* $Q=Q-\left\{i\in Q|d\left({ã}_{i}^{r}\right)=0\right\}$*.*
2. Search for i^*^ ∈ *Q as follows.*
  - *Let K* ⊂ *Q be the set of indices such that*
    $$\begin{array}{l} k=\arg\underset{i\in Q}{\max}d\left({ã}_{i}^{r}\right)\;\forall k\in K \end{array}$$
  - Among the entries of K, find i^*^ *such that*
    $$\begin{array}{l} i^{*}=\arg\underset{k\in K}{\max}T_{k}\left(P,Q\right) \end{array}$$
3. *Modify* (ã_*i*,*j*_) *and update P and Q as follows.*
  - Let u^*^ ∈ {0, 1} *be the value of* $x_{i^{*}}$ *such that* $T_{i^{*}}\left(P,Q\right)=|C_{i^{*}}\left(P;u^{*}\right)\cap Q|$*.*
  - *Set*
    $$\begin{array}{l} {ã}_{i^{*}}^{r}={ã}_{h}^{r}=0_{1\times n}\\ {ã}_{i^{*}}^{c}={ã}_{h}^{c}=0_{n\times 1}\;\forall h\in C_{i^{*}}\left(P;u^{*}\right)\cap Q \end{array}$$
  - *Update P and Q by*
    $$\begin{array}{l} P=P\cup \left\{\left(i^{*},u^{*}\right)\right\}\\ Q=Q-\left\{i^{*}\right\}\cup C_{i^{*}}\left(P;u^{*}\right) \end{array}$$
4. *If* (ã_*i*,*j*_) *is strictly upper triangular, terminate the algorithm. The solution to global stabilization of F is*
  $$\begin{array}{l} x_{i_{1}}=u_{1},\ldots ,x_{i_{\left|P\right|}}=u_{\left|P\right|} \end{array}$$
  *where P* = {(*i*_1_, *u*_1_), …, (*i*_|*P*|_, *u*_|*P*|_)}*. Otherwise, return to Step 2.*

Algorithm 2 is identical to Algorithm 1 except that (i) the column vector norm $\left|a_{j}^{c}\right|$ is employed instead of the row vector norm $\left|a_{i}^{r}\right|$ in permuting the adjacency matrix (Step 1), and (ii) $d\left({ã}_{i}^{r}\right)$, the number of 1's in off-diagonal entries of a row, replaces its column counterpart $d\left({ã}_{j}^{c}\right)$ in determining the control input (Step 2). Algorithm 1 is suitable for applying to BNs in which state variables with a large number of outgoing edges produce large canalization numbers. On the other hand, Algorithm 2 is pertinent to apply to BNs where state variables with a large number of incoming edges have a tendency to have large canalization numbers.

Example 5. *Let us apply Algorithm 2 to global stabilization of F in Example 1. We first permute* (*a*_*i*,*j*_) *according to an ascending order of the column vector norm. The permuted adjacency matrix* (ã_*i*,*j*_) *is shown in Table 2. After applying Step 2–4, we obtain P* = {(8, 1)}*, i.e., x*_8_ = 1 *as the control input that achieves global stabilization. Thus the control inputs and the global fixed point are the same as those derived using Algorithm 1 in Example 4.*

**Table 2:** Permuted adjacency matrix (ã_*i,j*_).

***x*_1_**	0	0	0	0	1	1	0	1
***x*_6_**	0	0	0	0	1	0	0	1
***x*_5_**	0	0	0	1	0	0	1	0
***x*_4_**	0	0	0	0	0	1	1	0
***x*_3_**	0	0	0	0	0	0	0	1
***x*_7_**	0	0	0	0	0	0	1	1
***x*_2_**	0	1	1	0	0	0	0	1
***x*_8_**	1	0	0	1	0	1	1	0
$\left|a_{j}^{c}\right|$	1	1	1	2	2	3	4	5

The proposed algorithm can be applied without modification to the case that some state variables serve as external inputs or outputs. We first remove input and output variables from the entries of the adjacency matrix. Then we derive the adjacency matrix by setting the values of external inputs and continue to apply Algorithm 1. The proposed algorithm is also applicable to the case that some state variables are disabled by mutation. For instance, if *x*_*i*_ is knocked out by mutation, its value is fixed to *x*_*i*_ = 0. In a similar way to Step 3.b of Algorithm 1, to deal with mutated variables we refine the adjacency matrix a priori by setting $a_{i}^{r}=a_{h}^{r}=0_{1\times n}$ and $a_{i}^{c}=a_{h}^{c}=0_{n\times 1}$ for all *h* ∈ [*n*] such that *x*_*h*_ is canalized by *x*_*i*_ = 0. Moreover, our algorithm can deal with the existence of uncontrollable state variables, namely those state variables that cannot be used as control inputs. Let *Q*_*f*_ ⊂ [*n*] be the index set of uncontrollable state variables. The latter constraint can be easily implemented in the algorithm by setting *Q*: = [*n*] − *Q*_*f*_ instead of *Q* = [*n*] in the initial phase.

As mentioned in Introduction, a significant advantage of the proposed algorithm is that it always guarantees a solution to global stabilization for any values of the external inputs and fixed values of mutated variables. Unless the external inputs and mutations influence the variables that are otherwise to be selected as control inputs, the algorithm gives the same solution without regard to the external inputs and mutations.

### 3.2. Sequential control

Once the heterogeneity of cellular responses is eliminated by the proposed scheme of global stabilization, it would be a reasonable follow-up measure to investigate whether there is a subsequent scheme that can drive the BN further from the unique fixed point to another stable state with a desirable feature. We may realize this objective by applying various control strategies for BNs (Cheng et al., 2011a; Kim et al., 2013; Mochizuki et al., 2013). In doing so, the contribution of our study to reduce the heterogeneity of the BN strewn with many mutations and input variations will play a role as an important precedence.

In this paper, we present one of straightforward subsequent schemes—to perturb the values of external inputs after the BN reaches the unique fixed point. We first assume that among *n* state variables of the considered BN, *m* ones (1 ≤ *m* < *n*) serve as external inputs, that is, they have no incoming edges in the corresponding influence graph. We also assume that some bio-markers of the cell are available to determine that the BN reaches an attractor and that a desirable phenotype turns on a specific combination of bio-markers. The proposed sequential control scheme combining global stabilization and perturbation of external inputs is addressed as follows.

*Step 1:* Given a BN *F*, apply Algorithm 1 (or Algorithm 2) to derive the set of constant control inputs *P* = {(*j*_1_, *u*_1_), …, (*j*_|*P*|_, *u*_|*P*|_)} that ensures global stabilization of *F*. Find the unique fixed point x^*^ ∈ {0, 1}^*n*^ that will be reached in response to *P*.
*Step 2:* Allocating x^*^ as the initial state, apply all 2^*m*^ input combinations to *F* separately during which |*P*| state variables *x*_*j*_1__, …, *x*_*j*_|*P*|__ that were used as control inputs are set to be free variables again.
*Step 3:* Check whether there exists an input combination that drives *F* from x^*^ to another fixed point with the desirable phenotype.
*Step 4:* If no such input combination is found, the sequential control scheme fails to achieve global stabilization to a desired fixed point. Else if there are a number of input combinations that succeed in favorable global stabilization, select the minimally perturbed input combination, namely, the input combination in which the number of activated external inputs is minimum.

As elucidated in Step 4, this control strategy does not always give a solution, as the reachability of the BN starting from the unique fixed point x^*^ may not be expanded enough by perturbing external inputs. Nevertheless, this method is worth attempting since it is very easy to apply and computationally tractable (usually the number of external inputs *m* is small). Once we derive the input combination that will be applied in the second step, we can conduct the overall procedure of the sequential control scheme as follows.

1. Provide *P* to the considered BN.
2. Determine the convergence of the BN to x^*^ by observing the corresponding bio-markers. When the convergence is ensured, stop the transmission of *P*.
3. Engage in the second control step by providing the input combination that is derived in the preceding algorithm.
4. Confirm the convergence of the BN to the desired fixed point by observing that the bio-markers change to the corresponding values.

The practicality of the sequential control scheme will be validated in our numerical experiments.

## 4. Application to biological systems

### 4.1. Metastasis influence network

**Figure 3:**
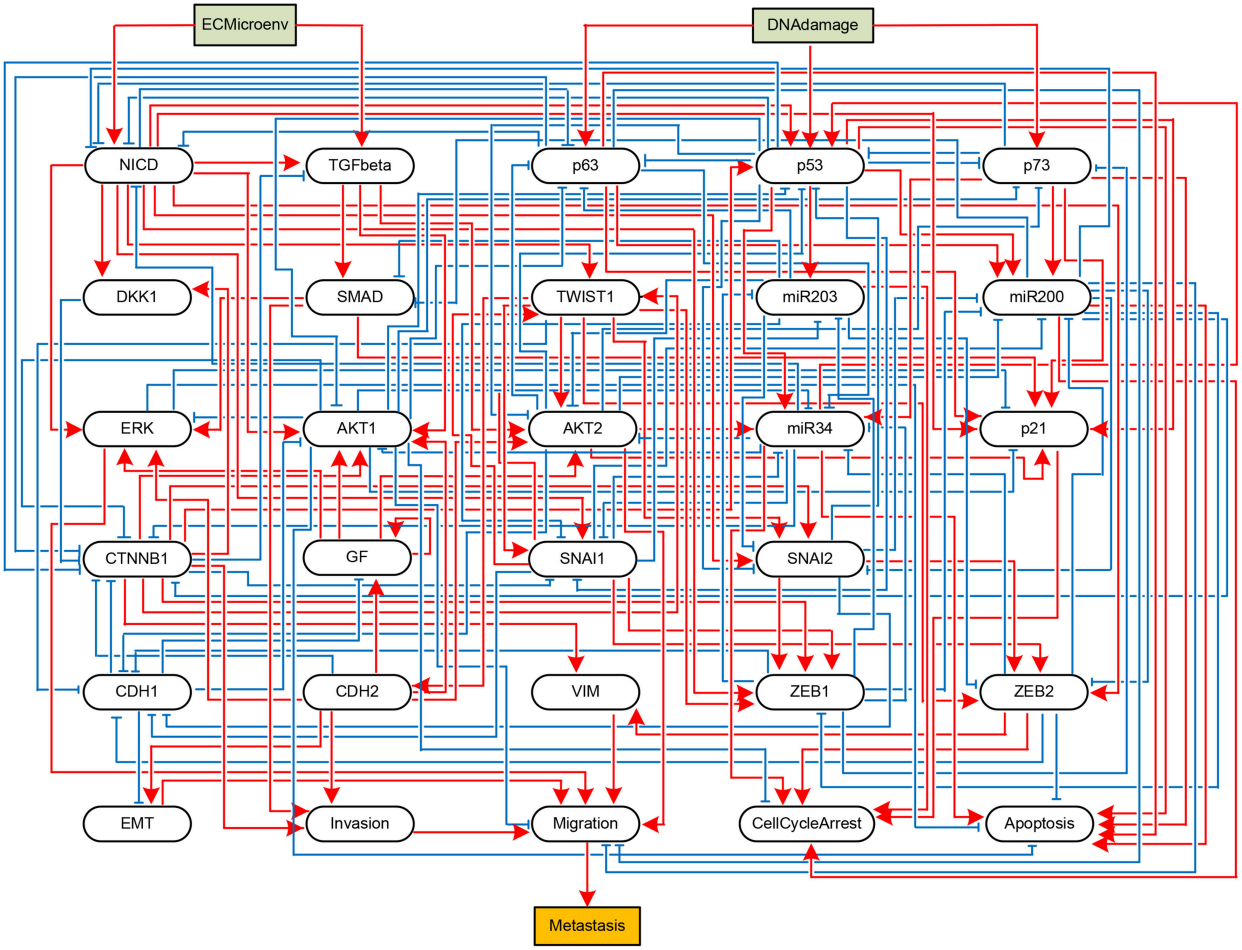
Boolean network implementing a metastasis influence network (Cohen et al., 2015). Some nodes represent biochemical species (proteins, miRNAs, processes, etc.) and others represent phenotypes, and edges represent activating (blue) or inhibitory (red) influences of one node onto other node. The BN has two input nodes *ECMicroenv* and *DNADamage* and one output node *Metastasis*, drawn in rectangles.

To validate the practicality of the proposed algorithm, we apply it to two real biological systems. First, let us consider an influence network describing the metastatic process of cells (Cohen et al., 2015). The network graph of the metastasis influence network is shown in Figure 3. There are two external inputs, *ECMicroenv* and *DNADamage*, and one output, *Metastasis*. *ECMicroenv* = 1 and 0 means that the effect of the extracellular micro-environment turns on and off, respectively. *DNADamage* = 1 implies that a DNA damage occurs to the considered cell. Excluding the inputs and output, the BN of Figure 3 has 29 free variables (*n* = 29). According to Cohen et al. (2015), it has nine possible fixed points in total.

Referring to Algorithm 1, we first compute the adjacency matrix (*a*_*i,j*_) and permute it to (ã_*i,j*_) according to the norm of the row vector. Display of (*a*_*i,j*_) and (ã_*i,j*_) is omitted here for space limit and the names of proteins shown in Figure 3 will take place of the corresponding indices. By Step 2, we derive $K=\left\{k\in Q|k=\arg\max d\left({ã}_{j}^{c}\right)\right\}$ where *Q* = [*n*] = {1, …, 29}. It turns out that *K* is a monotone set *K* = {*p*53}. Hence we have j^*^ = *p*53.

By Step 3.a, we replace *p53* with 0 and 1 respectively to the Boolean logic rules (Supplementary Table S1) to compute the canalization number. It is found that by setting *p53*=1, eight state variables *AKT1, AKT2, CTNNB1, NICD, p63, p73, SNAI1*, and *SNAI2* are maximally canalized to logic 0. For instance, the state transition equation of *AKT1* is (see Supplementary Table S1 and Cohen et al., 2015)

$$\begin{array}{l} AKT1=CTNNB1\wedge \left(NICD\vee TGFbeta\vee GF\vee CDH2\right)\\ \;\wedge \neg p53\wedge \neg miR34\wedge \neg CDH1 \end{array}$$

Since *p53* is included in the equation in the form “∧¬*p*53,” *p53* = 1 clearly leads to *AKT1* = 0.

Further, we investigate whether other variables are subsequently canalized by these first-canalized variables and so forth. Interestingly, all the other variables are canalized to fixed values. Therefore, we find that *p53* = 1 is a solution to global stabilization of the metastasis influence network (refer to Supplementary Dataset S4 for a Python script of Algorithm 1 for the Metastasis influence network). To confirm our result, we use a Python package called BooleanNet (Albert et al., 2008; BooleanNet, 2018) to search for attractors of the BN with the control input *p53* = 1 (see also the Supplementary Material BooleanNet). Table 3 is the outcome of the search given every possible combination of two inputs *DNADamage* and *ECMicroenv*. We ensure the validity of the proposed scheme since Table 3 equals the result of our scheme.

**Table 3:** Unique fixed points with *p53* = 1.

**Gene**	**attr1**	**attr2**	**attr3**	**attr4**
**DNADamage**	**0**	**0**	**1**	**1**
**ECMicroenv**	**0**	**1**	**0**	**1**
AKT1	0	0	0	0
AKT2	0	0	0	0
CDH1	1	1	1	1
CDH2	0	0	0	0
CTNNB1	0	0	0	0
DKK1	0	0	0	0
ERK	0	0	0	0
GF	0	0	0	0
miR200	1	1	1	1
miR203	1	1	1	1
miR34	0	0	0	0
NICD	0	0	0	0
p21	1	1	1	1
p53	1	1	1	1
p63	0	0	0	0
p73	0	0	0	0
SMAD	0	0	0	0
SNAI1	0	0	0	0
SNAI2	0	0	0	0
TGFbeta	0	1	0	1
TWIST1	0	0	0	0
VIM	0	0	0	0
ZEB1	0	0	0	0
ZEB2	0	0	0	0
CellCycleArrest	1	1	1	1
**Apoptosis**	**1**	**1**	**1**	**1**
EMT	0	0	0	0
Invasion	0	0	0	0
Migration	0	0	0	0
**Metastasis**	**0**	**0**	**0**	**0**

*The rows of two external inputs and one output, and the key gene, Apoptosis, are written in bold*.

An examination of Table 3 shows that four attractors attr1–attr4 are almost identical with each other (only the value of *TGFbeta* differs). Hence it can be said that the proposed scheme guarantees homogenous stable states of the considered BN against heterogeneity in terms of external inputs. Further, all the obtained attractors are desirable since they ensure programmed cell death and no metastasis is manifested (*Apoptosis* = 1 and *Metastasis* = 0 in all attractors). This result complies with the analysis in Cohen et al. (2015) that unless the mutation activating *NICD* and inhibiting *p53* occurs, the network will converge to apoptotic stable states.

Though any subsequent control is unnecessary in this case, we note that the proposed algorithm of global stabilization is not able to specify the features of the obtained fixed points in general since it does not use any parameter associated with the desirable phenotypes. Hence, if the obtained fixed points do not have desirable features, we must apply the second control step. The latter problem will be discussed in the next case study. We also note that Algorithm 2 produces the same result *p53* = 1 for this case study.

In Cohen et al. (2015), main consideration was devoted to constructing a logical model describing metastasis and to understanding the role of involved gene alterations. While some predictions were made on pathways and molecules triggering metastasis, no methodology was presented to determine control targets that can globally stabilize the metastasis influence network. Hence, our study can expedite further analysis of the metastasis influence network for control purposes.

### 4.2. Mapk signaling network

**Figure 4:**
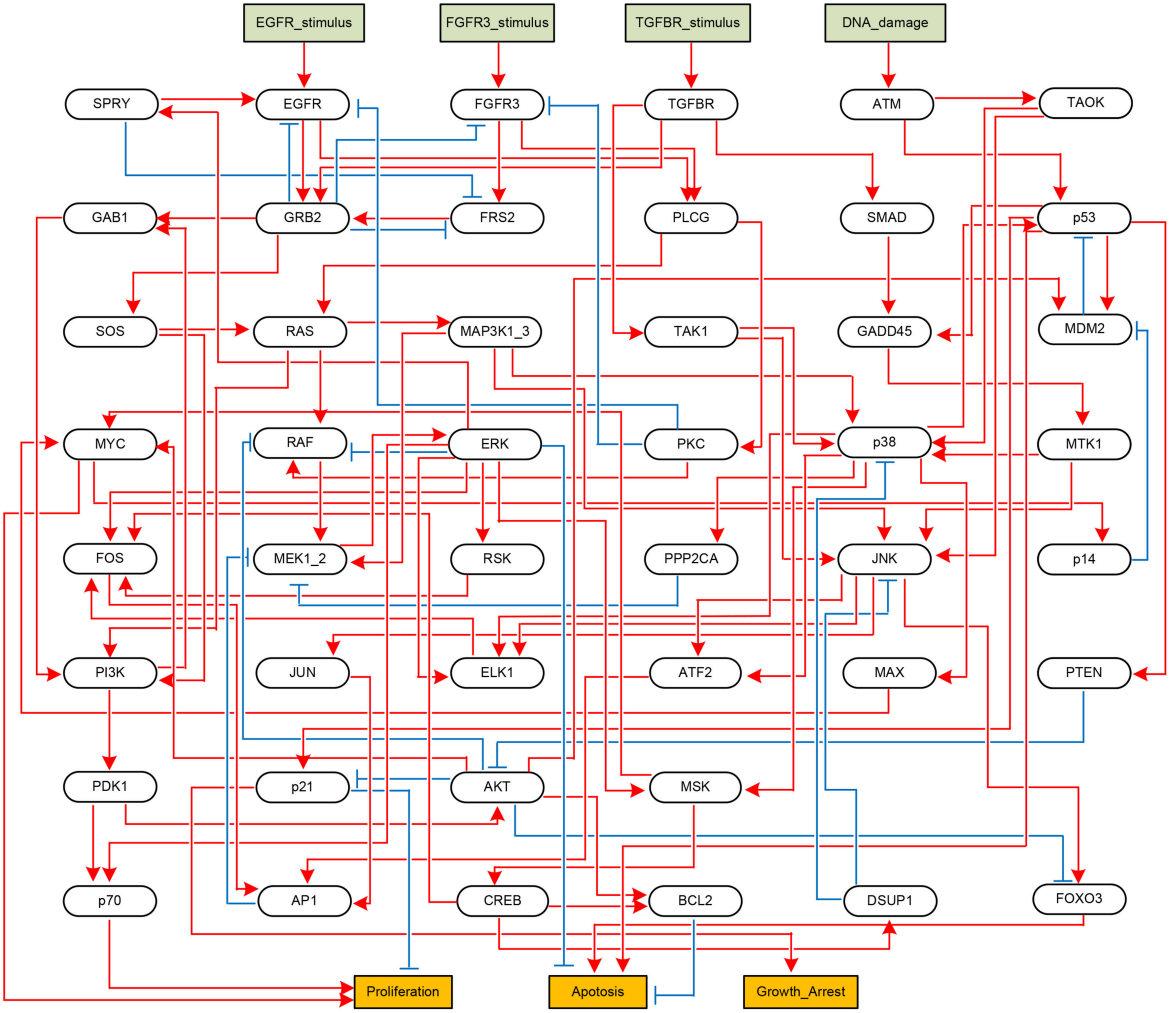
Boolean network implementing the MAPK signaling network (Grieco et al., 2013). Each node denotes a model component. Model inputs and outputs are drawn in rectangles, and blue arrows and red T-arrows denote positive and negative regulations, respectively.

Next, we apply the proposed algorithm to global stabilization of the Mitogen-activated protein kinase (MAPK) signaling network that describes the mechanism underlying the influence of the MAPK signaling network on cancer cell fate decision (Grieco et al., 2013). Represented as a BN shown in Figure 4, the MAPK signaling network has 53 components in total, among which there are four inputs (*DNA_damage, EGFR_stimulus, FGFR3_stimulus*, and *TGFBR_stimulus*) and three outputs (*Proliferation, Apoptosis*, and *Growth_Arrest*).

We have applied the proposed algorithm to the MAPK signaling network with various combinations of input sets and mutation settings, which are specified in Supplementary Dataset S3 of Grieco et al. (2013). For all the possible input combinations and mutation settings, our algorithm produces the same solution set, *p38* = 1 and *GRB2* = 1, that globally stabilizes the considered network (both Algorithms 1 and 2 derive the same result; refer to Supplementary Dataset S5 for a Python script of Algorithm 1). Table 4 is a list of selected results that shows attractors with respect to five combinations of external inputs and three mutation settings, denoted by r4, r9, and r10 following Grieco et al. (2013); refer to Supplementary Table S3 for attractors obtained with respect to all input combinations.

**Table 4:** Unique single attractors with *p38* = 1 and *GRB2* = 1 for various input combinations and mutation settings.

**Gene**	**Input set to 1**					**Mutation settings**		
	**None**	**DNA_damage**	**EGFR_stimulus**	**FGFR3_stimulus**	**TGFBR_stimulus**	**FGFR3 = 1(r4)**	**EGFR = 1p14 = 0(r9)**	**FGFR3 = 1p14 = 0(r10)**
AKT	0	0	0	0	0	0	1	1
AP1	1	1	1	1	1	1	0	0
ATF2	1	1	1	1	1	1	1	1
ATM	0	1	0	0	0	0	0	0
**Apoptosis**	**1**	**1**	**1**	**1**	**1**	**1**	**0**	**0**
BCL2	0	0	0	0	0	0	1	1
CREB	1	1	1	1	1	1	1	1
DUSP1	1	1	1	1	1	1	1	1
EGFR	0	0	0	0	0	0	1	0
ELK1	1	1	1	1	1	1	1	1
ERK	0	0	0	0	0	0	0	0
FGFR3	0	0	0	0	0	1	0	1
FOS	0	0	0	0	0	0	0	0
FOXO3	1	1	1	1	1	1	0	0
FRS2	0	0	0	0	0	0	0	0
GAB1	1	1	1	1	1	1	1	1
GADD45	1	1	1	1	1	1	0	0
GRB2	1	1	1	1	1	1	1	1
**Growth_Arrest**	**1**	**1**	**1**	**1**	**1**	**1**	**0**	**0**
JNK	1	1	1	1	1	1	0	0
JUN	1	1	1	1	1	1	0	0
MAP3K1_3	1	1	1	1	1	1	1	1
MAX	1	1	1	1	1	1	1	1
MDM2	0	0	0	0	0	0	1	1
MEK1_2	0	0	0	0	0	0	0	0
MSK	1	1	1	1	1	1	1	1
MTK1	1	1	1	1	1	1	0	0
MYC	1	1	1	1	1	1	1	1
PDK1	1	1	1	1	1	1	1	1
PI3K	1	1	1	1	1	1	1	1
PKC	0	0	0	0	0	1	1	1
PLCG	0	0	0	0	0	1	1	1
PPP2CA	1	1	1	1	1	1	1	1
PTEN	1	1	1	1	1	1	0	0
**Proliferation**	**0**	**0**	**0**	**0**	**0**	**0**	**0**	**0**
RAF	1	1	1	1	1	1	0	0
RAS	1	1	1	1	1	1	1	1
RSK	0	0	0	0	0	0	0	0
SMAD	0	0	0	0	1	0	0	0
SOS	1	1	1	1	1	1	1	1
SPRY	0	0	0	0	0	0	0	0
TAK1	0	0	0	0	1	0	0	0
TAOK	0	1	0	0	0	0	0	0
TGFBR	0	0	0	0	1	0	0	0
p14	1	1	1	1	1	1	0	0
p21	1	1	1	1	1	1	0	0
p38	1	1	1	1	1	1	1	1
p53	1	1	1	1	1	1	0	0
p70	0	0	0	0	0	0	0	0

*The rows of three genes composing the desirable phenotype are written in bold*.

This results imply a remarkable virtue of the proposed algorithm, i.e., despite differences in activations of the external inputs and mutation profiles, our scheme guarantees the global stabilization of the considered network. According to Supplementary Dataset S3 of Grieco et al. (2013), the number of attractors for each mutation setting is 3 for r4, 1 for r9, and 2 for r10. By contrast, applying the derived control inputs *p38* = 1 and *GRB2* = 1, we ensure that the network converges to a fixed point for any mutation profile. Moreover, as observed in Table 4, the global attractor for each case of the input combination and mutation setting is very similar to one another. For instance, in the attractors for all 2^4^ = 16 input combinations (among which only five are displayed in columns 2–6 of Table 4), only five state variables, *ATM, SMAD, TAK1, TAOK*, and *TGFBR*, have different values. The attractors obtained under mutation settings also have strong similarity with each other.

The reason for this similarity is obvious. Note that the proposed algorithm always searches for the target variables and corresponding values according to the number of outgoing edges and the canalization number (Algorithms 1 and 2). Since the latter values are little influenced by perturbation of external inputs, the result will be the same in most cases. Only state variables having incoming edges from external inputs or from those variables that are directly connected with external inputs will differ. In the above case, for example, *ATM* will vary according to the input *DNA_damage* since *ATM* = *DNA_damage* (see Supplementary Table S2).

Once heterogeneity of cellular responses is minimized by global stabilization, we can apply further control schemes to take the derived global attractor toward another attractor with desirable features. In this numerical experiment, we try to achieve this goal by perturbing four external inputs as presented in section 3.2. An apoptotic stable state of the MAPK signaling network is characterized by *Apoptosis* = *Growth_Arrest* = 1 and *Proliferation* = 0 (Grieco et al., 2013). Referring to Table 4, six left most attractors are apoptotic stable states while those of mutations r9 and r10 are not. We apply every combination of input perturbations to the two non-apoptotic attractors in order to conduct the second control. Note that in the second step, the foregoing control inputs *p38* = *GRB2* = 1 are not employed any more and *p38* and *GRB2* are released as free variables.

Table 5 shows the results of perturbation of external inputs after global stabilization for mutations r9 and r10 (see Supplementary Table S4 for complete description of attractors). Note that there are a number of input combinations among 16 candidates achieving the goal, namely, invoking the BN to reach apoptotic stable states in both mutations. We select the case of *DNA_damage* = *TGFBR_stimulus* = 1 as our solution since it needs the minimum number of input perturbations. The sequential control procedure for the MAPK signaling network for mutations r4, r9, and r10 is summarized as follows (see also section 3.2).

1. Apply control inputs *p38* = *GRB2* = 1 to drive the network toward the global fixed point of each mutation setting (Table 4 and Supplementary Table S3).
2. Determine the convergence of the network by observing the change of bio-markers (see Grieco et al., 2013).
3. Conduct the second control step by applying *DNA_damage* = *TGFBR_stimulus* = 1 so as to drive the network toward apoptotic attractors (Table 5 and Supplementary Table S4).

**Table 5:** Results of perturbation of external inputs after global stabilization by *p38* = 1 and *GRB2* = 1.

**Mutation setting**	**External inputs**				**Key genes in attractors**		
	**DNA_damage**	**EGFR_stimulus**	**FGFR3_stimulus**	**TGFBR_stimulus**	**Apoptosis**	**Growth_Arrest**	**Proliferation**
r9	0	0	0	0	Cycle	Cycle	Cycle
	0	0	0	1	0	0	0
	0	0	1	0	Cycle	Cycle	Cycle
	0	0	1	1	0	0	0
	0	1	0	0	Cycle	Cycle	Cycle
	0	1	0	1	0	0	0
	0	1	1	0	Cycle	Cycle	Cycle
	0	1	1	1	0	0	0
	1	0	0	0	1	1	0
	**1**	**0**	**0**	**1**	**1**	**1**	**0**
	1	0	1	0	1	1	0
	1	0	1	1	1	1	0
	1	1	0	0	1	1	0
	1	1	0	1	1	1	0
	1	1	1	0	1	1	0
	1	1	1	1	1	1	0
r10	0	0	0	0	Cycle	Cycle	Cycle
	0	0	0	1	0	0	0
	0	0	1	0	Cycle	Cycle	Cycle
	0	0	1	1	0	0	0
	0	1	0	0	Cycle	Cycle	Cycle
	0	1	0	1	0	0	0
	0	1	1	0	Cycle	Cycle	Cycle
	0	1	1	1	0	0	0
	1	0	0	0	Cycle	Cycle	Cycle
	**1**	**0**	**0**	**1**	**1**	**1**	**0**
	1	0	1	0	Cycle	Cycle	Cycle
	1	0	1	1	1	1	0
	1	1	0	0	Cycle	Cycle	Cycle
	1	1	0	1	1	1	0
	1	1	1	0	Cycle	Cycle	Cycle
	1	1	1	1	1	1	0

*The rows of the selected solution input combination are written in bold*.

Like the foregoing case study, the present result can contribute to determining control targets for the MAPK signaling network since the original study (Grieco et al., 2013) did not consider the latter topic. The major concern of Grieco et al. (2013) was to present a logical model of the MAPK signaling network and to elucidate how MAPK signaling affects cell proliferation, growth arrest, apoptotic cell death, etc. Grieco et al. (2013) applied known biological input/output data to the MAPK signaling network, based on which the underlying mechanisms were analyzed in detail. Note that such analysis was focused on understanding the mechanism with respect to feedbacks and cross-talks inherent in the model, not on determining control targets for global stabilization as done in this study.

As mentioned, the proposed algorithm cannot specify the feature of the unique fixed point in a desirable way, which may impose a burden on the second control step. But our sequential control scheme can still be useful, especially in controlling cancer cells, for the following reasons:

- First, for cancer cells, removing non-genetic heterogeneity via global stabilization is a very significant phase itself that should be achieved even though the resulting attractor is unsatisfactory. Sequential control of cancer cells, i.e., initially blocking primary mutation effects and cross-talks, and subsequently applying combinatorial targeted drugs for additional control, has been an active area of research in recent years [see, e.g., Lee et al. (2012); Vijayaraghavalu et al. (2012)].
- Next, while many existing targeted drugs aim at inhibiting or activating intracellular molecules (mainly signaling proteins) of cancer cells, studies on tackling cancer cells by manipulating tumor micro-environments are also receiving a great attention. Since tumor micro-environments are characterized by external inputs in BNs, our sequential control scheme with external input control in the second step can be combined with the related methods [e.g., Bissell and Hines (2011); Quail and Joyce (2013)].

### 4.3. Comparative study

To conduct a comparative study, we have applied three representative global stabilization schemes—feedback vertex set (FVS) control (Fiedler et al., 2013), the control kernel (CK) method (Kim et al., 2013), and the stable motif (SM) method (Zañudo and Albert, 2015) to the control problem of the MAPK signaling network discussed in the previous subsection.

**(i) Feedback vertex set control:** In graph theory, an FVS is a subset of nodes in the absence of which the digraph becomes acyclic, i.e., it contains no directed cycles (Fiedler et al., 2013; Liu and Barabàsi, 2016). Hence if constant control inputs are assigned to the state variables of an FVS, the resultant BN will eventually converge to a unique fixed point. To apply FVS control, we first identify a desired fixed point that is possessed by the considered BN, namely, a fixed point showing the desirable phenotype (*Apoptosis* = *Growth_Arrest* = 1 and *Proliferation* = 0). To this end, we randomly generated 100,000 initial states and made the MAPK signaling network evolve from each initial state. In this near brute-force searching, we found eight fixed points for r9 mutation and four ones for r10 mutation that have the desirable phenotype. We select a desired fixed point from each attractor set for r9 and r10 mutations, respectively, and derive the minimal FVS. Then we set the values of FVS according to the corresponding values in the selected fixed point. Although some cyclic attractors also have the desirable phenotype, we did not use them for the purpose of focusing on fixed points.

Referring to Supplementary Dataset S1, we found that Attractor 2 of r9 mutation and Attractor 21 of r10 mutation are the same. Hence by selecting this fixed point, we can achieve global stabilization of the BN with desirable phenotype irrespective of the existence of both r9 and r10 mutations. Table 6 shows the minimal FVSs that take the MAPK signaling network toward the desired fixed point. The result of Table 6 is similar to that of Table 5 in that both solve the global stabilization problem by activating two external inputs (*DNA_damage* and *TGFBR_stimulus*) and by setting some state variables to be constant controls.

**Table 6:** Minimal FVS for control of the MAPK signaling network with r9 and r10 mutations (refer to Supplementary Dataset S1 for associated desired fixed points).

**External inputs**				**Internal variables**					
**DNA_damage**	**EGFR_stimulus**	**FGFR3_stimulus**	**TGFBR_stimulus**	**ERK**	**p53**	**p38**	**PKC**	**GRB2**	**GAB1**
1	0	0	1	0	1	1	1	1	1

In term of accessibility of the modeling information, FVS control is superior since it does not need the exact Boolean logic of the BN. On the other hand, the solution of the proposed scheme is more efficient in this numerical experiment since the number of control inputs is less than that of FVS control. In fact, our solution set *p38* = 1 and *GRB2* = 1 is included in the minimal FVS as seen in Table 6.

**(ii) Control kernel method:** In Kim et al. (2013), the control kernel is defined as the minimal set of nodes that need to be regulated to drive the network to converge to a desired attractor for all initial states. A genetic algorithm (GA) is employed to find the minimal set among randomly selected candidate node sets. Following the method addressed in Kim et al. (2013), we found the control kernel that drives the MAPK signaling network to a fixed point with the desirable phenotype (*Apoptosis* = *Growth_Arrest* = 1 and *Proliferation* = 0). Since global stabilization must be valid for either r9 or r10 mutation, we searched for the control kernel for each mutation case separately and extracted common control kernels, if any. The control kernel method usually chooses a desired fixed point, based on which an appropriate control kernel is explored. But we did not specify any desired fixed point in this case study. Instead, we adapted the control kernel algorithm and discovered feasible control kernels and their corresponding fixed points yielding the desirable phenotype simultaneously in the search space of the control kernel method.

It is found that no control kernel having size one exists that achieves global stabilization of the BN. With the size of the control kernel set to be two, we found nine control kernels that solve the control problem, as shown in Table 7 and Supplementary Dataset S2. Interestingly, our solution set *p38* = 1 and *GRB2*=1 is not included in the derived control kernels. This is due to the property of our adapted searching algorithm that it explores the control kernel and a desired attractor in one single step. The result of Table 7 indicates that the control kernel has superior performance than the proposed scheme since it does not need any external input to be activated. In term of computational load, however, our algorithm is much better since while the control kernel method takes more than 72 h to obtain the result, our algorithm yields the control inputs and the associated external inputs in a few minutes.

**Table 7:** Control kernels with size two for control of the MAPK signaling network with r9 and r10 mutations (refer to Supplementary Dataset S2 for associated desired fixed points).

	**External inputs**				**Internal variables**					
	**DNA_damage**	**EGFR_stimulus**	**FGFR3_stimulus**	**TGFBR_stimulus**	**ATM**	**FRS2**	**GRB2**	**TGFBR**	**p53**	**MDM2**
1	-	-	-	-	1	1	-	-	-	-
2	-	-	-	-	1	-	1	-	-	-
3	-	-	-	-	1	-	-	1	-	-
4	-	-	-	-	-	1	-	-	-	1
5	-	-	-	-	-	1	-	-	1	-
6	-	-	-	-	-	-	1	-	-	1
7	-	-	-	-	-	-	1	-	1	-
8	-	-	-	-	-	-	-	1	-	1
9	-	-	-	-	-	-	-	1	1	-

“*-” indicates that the corresponding variable or external input is not needed*.

**(iii) Stable motif method:** A stable motif is referred to as a set of nodes and their corresponding states such that the nodes form a minimal strongly connected component and their states form a partial fixed point of the BN (Zañudo and Albert, 2015). Stable motifs can be regarded as control targets since once they reach certain Boolean values, they are preserved against other updating schemes due to their dynamical property of being partial fixed points. In Zañudo and Albert (2015), the set of stable motifs is first computed, followed by reducing the number of control targets in the stable motif using the *stable motif control algorithm*. The stable motif method is remarkable since it is the first network control approach that combines the structural and functional information of Boolean networks to determine control inputs for stabilization.

We have applied the stable motif method to controlling the MAPK signaling network that is influenced by r9 and r10 mutations. The stable motif control algorithm was implemented based on the method of Zañudo et al. (2017), and StableMotifs java library devised in Zañudo and Albert (2015) was used to realize the simulation code. It is found that the set of stable motifs for each mutation profile contains more than 10 state variables. However, through the stable motif control algorithm, we derived a number of stable motif control sets consisting of only four external inputs (Supplementary Dataset S3). Among them, four combinations shown in Table 8 globally stabilize the BN to a fixed point having the desirable phenotype (*Apoptosis* = *Growth_Arrest* = 1 and *Proliferation* = 0) for both r9 and r10 mutations.

**Table 8:** Stable motif control sets for control of the MAPK signaling network with r9 and r10 mutations (refer to Supplementary Dataset S3 for associated desired fixed points).

	**External inputs**			
	**DNA_damage**	**EGFR_stimulus**	**FGFR3_stimulus**	**TGFBR_stimulus**
1	1	0	0	1
2	1	0	1	1
3	1	1	0	1
4	1	1	1	1

The result of the stable motif method is efficient in that the solution set includes no internal variables. Hence it would be more advantageous when manipulating control targets in biological experiments. On the other hand, it is necessary to identify all attractors of the MAPK signaling network before determining the stable motif control set that will be utilized as actual controls since some quasi-attractors induced in the network reduction procedure are not real attractors of the BN (see Supplementary Dataset S3).

The primary difference between the proposed scheme and the existing methods is that the proposed scheme is a purely analytical approach for solving the global stabilization problem based on structural and algebraic information of the BN. The proposed scheme is particularly useful for large-scale biological networks as it does not involve any numerical search algorithm with demanding complexity. Table 9 summarizes our comparative study with a brief review of the procedure of each control scheme.

**Table 9:** Comparison between the proposed scheme and feedback vertex set, control kernel, and stable motif methods that are applied to controlling the MAPK signaling network.

	**Proposed scheme**	**Feedback vertex set**	**Control kernel**	**Stable motif**
Find control targets for global stabilization	Yes	Yes	Yes	Yes
Applicable to large-scale BNs (*n* ≥ 100)	Yes	Yes	Yes^†^	No
Need to know Boolean logic of the network	Yes	No	Yes	Yes
Procedure	1. Global stabilization by the adjacency matrix2. Determine external inputs to steer the BN toward a desired attractor	1. Find FVSs using network topology2. Fix values of FVS states corresponding to the desired attractor	Check whether the BN can be steered toward the desired attractor by brute-force method (sample initial states for large networks)	1. Compute stable motifs2. Derive optimal stable motif nodes that take the BN to the desired attractor

† *Note that the control kernel method is computationally intractable, if not impossible, for large-scale BNs since it takes huge time to find control kernels for BNs with large n*.

## 5. Conclusion

The problem of global stabilization of BNs has been addressed in this paper to control the heterogeneous cellular behavior for homogeneous responses. We have proposed an algorithm determining a set of constant control inputs that can drive the controlled BN to an unspecified global fixed point. A subsequent control to transform the fixed point to a desired attractor is further presented using perturbation of external inputs. The proposed sequential control method is practical in that the procedure of selecting control inputs is simple and has polynomial computational complexity with respect to the dimension of state variables, while having exponential complexity with respect to in-degree of BNs. In addition, the proposed method can be used for any combination of external inputs and mutations. The results of numerical experiments on the metastasis regulation influence network and MAPK signaling network demonstrate the applicability of the proposed control scheme. Furthermore, our experimental studies show that the proposed sequential control can drive the BN to reach a desired final attractor and the proposed global stabilization can be utilized as a preparatory step.

## Author contributions

J-MY devised the algorithm and implemented it. C-KL worked on simulation analysis. K-HC designed the project and supervised the study. J-MY and K-HC wrote the manuscript.

### Conflict of interest statement

The authors declare that the research was conducted in the absence of any commercial or financial relationships that could be construed as a potential conflict of interest.

## Supplementary material

Supplementary information on the two biological Boolean network models, the details of the proposed control targets, and the implementation information and results of comparative studies are provided:

Supplementary Table S1 Boolean logical rules describing the activity of nodes in the Metastasis influence network.

Supplementary Table S2 Boolean logical rules describing the activity of nodes in the MAPK signaling network.

Supplementary Table S3 Unique single attractors with *p38* = 1 and *GRB2* = 1 for all the possible input combinations in the MAPK signaling network.

Supplementary Table S4 Complete description of the attractors in Table 4 that are obtained by perturbation of external inputs after global stabilization by *p38* = 1 and *GRB2* = 1.

Supplementary Dataset S1 Results of FVS control for the MAPK signaling network.

- “r9_RealAttractors” sheet: All the attractors of the BN with r9 mutation that are obtained by randomly generating 100,000 initial states.
- “r10_RealAttractors” sheet: All the attractors of the BN with r10 mutation that are obtained by randomly generating 100,000 initial states.
- “Minimal FVSs” sheet: Derived minimal FVSs with respect to Attractor 2 in r9_RealAttractors and Attractor 21 in r10_RealAttractors sheets.
- “Results” sheet: Selected minimal FVSs and the desired fixed points for r9 and r10 mutations.

Supplementary Dataset S2 Results of the control kernel method for the MAPK signaling network.

- “r9_CKs” sheet: All the control kernels with size two that stabilize the BN with r9 mutation to a set of desired fixed points.
- “r10_CKs” sheet: All the control kernels with size two that stabilize the BN with r10 mutation to a set of desired fixed points.
- “Results” sheet: Intersection of the control kernels with size two for r9 and r10 mutations.

Supplementary Dataset S3 Results of the stable motif method for the MAPK signaling network.

- “r9_RealAttractors” sheet: All the attractors of the BN with r9 mutation that are obtained by randomly generating 100,000 initial states.
- “r10_RealAttractors” sheet: All the attractors of the BN with r10 mutation that are obtained by randomly generating 100,000 initial states.
- “r9_StableMotifControlSets” sheet: Stable motif control sets of the BN with r9 mutation that are obtained by the stable motif control algorithm.
- “r10_StableMotifControlSets” sheet: Stable motif control sets of the BN with r10 mutation that are obtained by the stable motif control algorithm.
- “r9_and_r10_QuasiAttractors” sheet: Quasi-attractors of the BN with r9 and r10 mutations that are obtained by the stable motif control algorithm.
- “r9_StableMotifControl_results” sheet: Desired fixed points of the BN with r9 mutation that are obtained by applying common stable motif control sets.
- “r10_StableMotifControl_results” sheet: Desired fixed points of the BN with r10 mutation that are obtained by applying common stable motif control sets.

Supplementary Dataset S4 A Python script that conducts global stabilization by Algorithm 1 for the Metastasis influence network (section 4.1). It can be also downloaded from https://github.com/choonlog/Global-stabilization/tree/master/GS_Adjacency_Matrix.

Supplementary Dataset S5 A Python script that conducts global stabilization by Algorithm 1 for the MAPK signaling network (section 4.2). It can be also downloaded from https://github.com/choonlog/Global-stabilization/tree/master/GS_Adjacency_Matrix.

BooleanNet A Python package called BooleanNet executed on Python 3.5 is used in searching for attractors in all the numerical experiments. By setting simulation parameters of the initial state of each node, logical functions of the BN, limit of trajectory steps, and the update scheme, we can find associated attractors. Notice that we have modified the source code of BooleanNet for enhancing computing efficiency. Download distribution is provided in https://github.com/choonlog/Global-stabilization.
