# Supplementary material for: Global Stabilization of Boolean Networks to Control the Heterogeneity of Cellular Responses
Source: Front Physiol. 2018 Jul 17;9:774. doi: 10.3389/fphys.2018.00774 (PMC6060448; doi:10.3389/fphys.2018.00774)
Supplement: Supplementary Table S1 — Boolean logical rules describing the activity of nodes in the Metastasis influence network. [file Table_1.PDF]

**Supplementary Table S1**: Boolean logical rules describing the activity of nodes in the Metastasis influence network (Cohen et al., 2015)

<table>
<tr><th>Node</th><th>Rule</th></tr>
<tr><td>AKT1</td><td>CTNNB1 & (NICD | TGFbeta | GF | CDH2) & !p53 & !miR34 & !CDH1</td></tr>
<tr><td>AKT2</td><td>TWIST1 & (TGFbeta | GF | CDH2) & !(miR203 | miR34 | p53)</td></tr>
<tr><td>CDH1</td><td>!TWIST1 & !SNAI2 & !ZEB1 & !ZEB2 & !SNAI1 & !AKT2</td></tr>
<tr><td>CDH2</td><td>TWIST1</td></tr>
<tr><td>CTNNB1</td><td>!DKK1 & !p53 & !AKT1 & !miR34 & !miR200 & !CDH1 & !CDH2 & !p63</td></tr>
<tr><td>DNAdamage</td><td>*Input*</td></tr>
<tr><td>DKK1</td><td>CTNNB1 | NICD</td></tr>
<tr><td>ERK</td><td>(SMAD | CDH2 | GF | NICD) & !AKT1</td></tr>
<tr><td>ECMicroenv</td><td>*Input*</td></tr>
<tr><td>GF</td><td>!CDH1 & (GF | CDH2)</td></tr>
<tr><td>miR200</td><td>(p63 | p53 | p73) & !(AKT2 | SNAI1 | SNAI2 | ZEB1 | ZEB2)</td></tr>
<tr><td>miR203</td><td>p53 & !(SNAI1 | ZEB1 | ZEB2)</td></tr>
<tr><td>miR34</td><td>!(SNAI1 | ZEB1 | ZEB2) & (p53 | p73) & AKT2 & !p63 & !AKT1</td></tr>
<tr><td>NICD</td><td>!p53 & !p63 & !p73 & !miR200 & !miR34 & ECM</td></tr>
<tr><td>p21</td><td>((SMAD & NICD) | p63 | p53 | p73 | AKT2) & !(AKT1 | ERK)</td></tr>
<tr><td>p53</td><td>(DNAdamage | CTNNB1 | NICD | miR34) & !SNAI2 & !p73 & !AKT1 & !AKT2</td></tr>
<tr><td>p63</td><td>DNAdamage & !NICD & !AKT1 & !AKT2 & !p53 & !miR203</td></tr>
<tr><td>p73</td><td>DNAdamage & !p53 & !ZEB1 & !AKT1 & !AKT2</td></tr>
<tr><td>SMAD</td><td>TGFbeta & !miR200 & !miR203</td></tr>
<tr><td>SNAI1</td><td>(NICD | TWIST1) & !miR203 & !miR34 & !p53 & !CTNNB1</td></tr>
<tr><td>SNAI2</td><td>(TWIST1 | CTNNB1 | NICD) & !miR200 & !p53 & !miR203</td></tr>
<tr><td>TGFbeta</td><td>(ECM | NICD) & !CTNNB1</td></tr>
<tr><td>TWIST1</td><td>CTNNB1 | NICD | SNAI1</td></tr>
<tr><td>VIM</td><td>CTNNB1 | ZEB2</td></tr>
<tr><td>ZEB1</td><td>((TWIST1 & SNAI1) | CTNNB1 | SNAI2 | NICD) & !miR200</td></tr>
<tr><td>ZEB2</td><td>(SNAI1 | (SNAI2 & TWIST1) | NICD) & !miR200 & !miR203</td></tr>
<tr><td>CellCycleArrest</td><td>(miR203 | miR200 | miR34 | ZEB2 | p21) & !AKT1</td></tr>
<tr><td>Apoptosis</td><td>(p53 | p63 | p73 | miR200 | miR34) & !ZEB2 & !AKT1 & !ERK</td></tr>
<tr><td>EMT</td><td>CDH2 & !CDH1</td></tr>
<tr><td>Invasion</td><td>(SMAD & CDH2) | CTNNB1</td></tr>
<tr><td>Migration</td><td>VIM & AKT2 & ERK & !miR200 & !AKT1 & EMT & Invasion & !p63</td></tr>
<tr><td>Metastasis</td><td>Migration</td></tr>
</table>
