# Supplementary material for: Global Stabilization of Boolean Networks to Control the Heterogeneity of Cellular Responses
Source: Front Physiol. 2018 Jul 17;9:774. doi: 10.3389/fphys.2018.00774 (PMC6060448; doi:10.3389/fphys.2018.00774)
Supplement: Supplementary Table S2 — Boolean logical rules describing the activity of nodes in the MAPK signaling network. [file Table_2.PDF]

Supplementary Table S2: Boolean logical rules describing the activity of nodes in the MAPK signaling network (Grieco et al., 2013)

**Node**	**Rule**
AKT	PDK1 & !PTEN
AP1	JUN & (FOS | ATF2)
Apoptosis	!BCL2 & !ERK & FOXO3 & p53
ATF2	JNK | p38
ATM	DNA_damage
BCL2	CREB & AKT
CREB	MSK
DNA_damage	*Input*
DUSP1	CREB
EGFR	(EGFR_stimulus | SPRY) & !(PKC | GRB2)
EGFR_stimulus	*Input*
ELK1	ERK | JNK | p38
ERK	MEK1_2
FGFR3	FGFR3_stimulus & !(GRB2 | PKC)
FGFR3_stimulus	*Input*
FOS	ERK & RSK & (ELK1 | CREB)
FOXO3	JNK & !AKT
FRS2	FGFR3 & !SPRY & !GRB2
GAB1	GRB2 | PI3K
GADD45	SMAD | p53
GRB2	EGFR | FRS2 | TGFBR
Growth_Arrest	p21
JNK	(TAOK & MAP3K1_3) | (MAP3K1_3 & MTK1) | (TAOK & MTK1) | (TAK1 & MTK1) | (TAK1 & MAP3K1_3) | (TAK1 & TAOK) | ((TAOK | MTK1 | MAP3K1_3 | TAK1) & !DUSP1)
JUN	JNK
MAP3K1_3	RAS
MAX	p38
MDM2	(p53 | AKT) & !p14
MEK1_2	(RAF | MAP3K1_3) & !(PPP2CA | AP1)
MSK	ERK | p38
MTK1	GADD45
MYC	(MSK & MAX) | (MSK & AKT)
p14	MYC
p21	!AKT & p53
p38	(TAOK & MAP3K1_3) | (MAP3K1_3 & MTK1) | (TAOK & MTK1) | (TAK1 & MTK1) | (TAK1 & MAP3K1_3) | (TAK1 & TAOK) | ((TAOK | MTK1 | MAP3K1_3 | TAK1) & !DUSP1)
p53	(ATM & p38) | ((ATM | p38) & !MDM2)

p70	PDK1 & ERK
PDK1	PI3K
PI3K	GAB1 | (RAS & SOS)
PKC	PLCG
PLCG	EGFR | FGFR3
PPP2CA	p38
Proliferation	p70 & MYC & !p21
PTEN	p53
RAF	(RAS | PKC) & !(ERK | AKT)
RAS	SOS | PLCG
RSK	ERK
SMAD	TGFBR
SOS	GRB2 & !RSK
SPRY	ERK
TAK1	TGFBR
TAOK	ATM
TGFBR	TGFBR_stimulus
TGFBR_stimulus	*Input*
