# Supplementary material for: Global Stabilization of Boolean Networks to Control the Heterogeneity of Cellular Responses
Source: Front Physiol. 2018 Jul 17;9:774. doi: 10.3389/fphys.2018.00774 (PMC6060448; doi:10.3389/fphys.2018.00774)
Supplement: Supplementary Dataset S4 — A Python script that conducts global stabilization by Algorithm 1 for the Metastasis influence network (section 4.1). It can be also downloaded from https://github.com/choonlog/Global-stabilization/tree/master/GS_Adjacency_Matrix. [file Data_Sheet_4.pdf]

GlobalStablization_Mestasis_influence_network.py

```
"""
GlobalStablization
Author : Jung-Min Yang(Kyungpook National University), Chun-Kyung Lee(Kyungpook National University), and Kwang-Hyun Cho(Korea Advanced Institute of Science and Technology)
"""
import itertools
import re
import operator
import time

start_time = time.time()
state = {}
nodeUpdate = {}
input = {}
resultSet = {}
inputCombiNum = 0

def canal(lineText):

    lineTextSplit = lineText.split("=")
    leftNode = lineTextSplit[0].strip()
    address = lineTextSplit[1].strip()
    lineText = lineText.replace('(', '').replace(')', '').replace('=', '')
    fullNode = []
    node = []
    duple = []
    nodeDic = {}
    u = 0

    if state[leftNode] == "True" or state[leftNode] == "False":
        return False
    else:
        for x in lineText.split():
            if(x not in ['or', 'and', 'not']):
                fullNode.append(x)

        for j in range(1, len(fullNode)):

            if state[fullNode[j]] == "True":
                nodeDic[fullNode[j]] = "True"
            elif state[fullNode[j]] == "False":
                nodeDic[fullNode[j]] = "False"
            else:
                node.append(fullNode[j])

        combinationNumber = len(node);
        for subset1 in itertools.combinations(node, combinationNumber):
            for k in range(0, combinationNumber + 1):
                for subset2 in itertools.combinations(subset1, k):
                    nodeOnList = [i for i in subset2]
                    nodeOffList = list(subset1)
                    for j in nodeOnList:
```

```
                        nodeOffList.remove(j)
                    for i in nodeOnList:
                        nodeDic[i] = "True"
                    for i in nodeOffList:
                        nodeDic[i] = "False"

                    result = eval(re.sub(r"₩b(₩S+)₩b", lambda m: nodeDic.get(m.group(1), m.group(1)), address))
                    duple.append(result)
                    if u > 0:
                        if duple[0] != duple[u]:
                            break;
                    u += 1

        removedDuple = [duple[i] for i in range(len(duple)) if i == duple.index(duple[i])]

        if len(removedDuple) == 1:
            nodeUpdate[leftNode] = removedDuple[0]

def iteration(modeltext):
    global nodeUpdate
    global state
    step = True

    while step:
        modeltextLine = modeltext.splitlines()
        for m in modeltextLine:
            if not (m == ""):
                canal(m)

        if len(nodeUpdate) > 0:
            for v in nodeUpdate:
                state[v] = str(nodeUpdate[v])
            nodeUpdate = {}
        else:
            step = False

    return(state)
    state = {}

def set(modeltext, input):

    for w in input:
        state[w] = input[w]

    left = {}
    leftOrder = []
    logic = []
    all = []

    modeltextLine = modeltext.splitlines()
    for m in modeltextLine:
        if not (m == ""):
```

```
            lineTextSplit = m.split("=")
            leftOrder.append(lineTextSplit[0].strip())
            left[lineTextSplit[0].strip()] = 0
            logic.append(lineTextSplit[1].strip())

    for j in left:
        for i in logic:
            if i.count(j) > 0:
                left[j] += 1
            else:
                left[j] += 0

    sorted_x = sorted(left.items(), key=operator.itemgetter(1), reverse=True)
    rankLeft = []

    for x in sorted_x:
        rankLeft.append(x[0])
    for g in input:
        rankLeft.remove(g)
        leftOrder.remove(g)

    del rankLeft[5:]

    e = 1
    while e < 3:
        combinationNumber = e;
        for subset1 in itertools.combinations(rankLeft, combinationNumber):
            for k in range(0, combinationNumber + 1):
                for subset2 in itertools.combinations(subset1, k):
                    leftOffList = [i for i in subset2]
                    leftOnList = list(subset1)
                    for j in leftOffList:
                        leftOnList.remove(j)
                    for q in leftOrder:
                        if q in leftOnList:
                            state[q] = "True"
                        elif q in leftOffList:
                            state[q] = "False"
                        else:
                            state[q] = ""

                    result = iteration(modeltext)
                    z = 0
                    for l in result:
                        if result[l]:
                            z += 1
                    if len(left) == z:
                        all.append({"On": leftOnList, "Off": leftOffList})
        e += 1
    return (all)

modeltext = '''
Metastasis = Migration
```

```
Migration = VIM and AKT2 and ERK and not miR200 and not AKT1 and EMT and Invasion and not p63
Invasion = (SMAD and CDH2) or CTNNB1
EMT = CDH2 and not CDH1
Apoptosis = (p53 or p63 or p73 or miR200 or miR34) and not ZEB2 and not AKT1 and not ERK
CellCycleArrest = (miR203 or miR200 or miR34 or ZEB2 or p21) and not AKT1
ECM = ECM
DNAdamage = DNAdamage
GF = not CDH1 and (GF or CDH2)
TGFbeta = (ECM or NICD) and not CTNNB1
p21 = ((SMAD and NICD) or p63 or p53 or p73 or AKT2) and not (AKT1 or ERK)
CDH1 = not TWIST1 and not SNAI2 and not ZEB1 and not ZEB2 and not SNAI1 and not AKT2
CDH2 = TWIST1
VIM = CTNNB1 or ZEB2
TWIST1 = CTNNB1 or NICD or SNAI1
SNAI1 = (NICD or TWIST1) and not miR203 and not miR34 and not p53 and not CTNNB1
SNAI2 = (TWIST1 or CTNNB1 or NICD) and not miR200 and not p53 and not miR203
ZEB1 = ((TWIST1 and SNAI1) or CTNNB1 or SNAI2 or NICD) and not miR200
ZEB2 = (SNAI1 or (SNAI2 and TWIST1) or NICD) and not miR200 and not miR203
AKT1 = CTNNB1 and (NICD or TGFbeta or GF or CDH2) and not p53 and not miR34 and not CDH1
DKK1 = CTNNB1 or NICD
CTNNB1 = not DKK1 and not p53 and not AKT1 and not miR34 and not miR200 and not CDH1 and not
CDH2 and not p63
NICD = not p53 and not p63 and not p73 and not miR200 and not miR34 and ECM
p63 = DNAdamage and not NICD and not AKT1 and not AKT2 and not p53 and not miR203
p53 = (DNAdamage or CTNNB1 or NICD or miR34) and not SNAI2 and not p73 and not AKT1 and not
AKT2
p73 = DNAdamage and not p53 and not ZEB1 and not AKT1 and not AKT2
miR200 = (p63 or p53 or p73) and not (AKT2 or SNAI1 or SNAI2 or ZEB1 or ZEB2)
miR203 = p53 and not (SNAI1 or ZEB1 or ZEB2)
miR34 = not (SNAI1 or ZEB1 or ZEB2) and (p53 or p73) and AKT2 and not p63 and not AKT1
AKT2 = TWIST1 and (TGFbeta or GF or CDH2) and not (miR203 or miR34 or p53)
ERK = (SMAD or CDH2 or GF or NICD) and not AKT1
SMAD = TGFbeta and not miR200 and not miR203
'''
inputSet = ["ECM", "DNAdamage"]

for f in range(0, len(inputSet) + 1):
    for subset in itertools.combinations(inputSet, f):
        nodeOnList = [d for d in subset]
        nodeOffList = list(inputSet)
        for t in nodeOnList:
            nodeOffList.remove(t)
        for y in nodeOnList:
            input[y] = "True"
        for y in nodeOffList:
            input[y] = "False"
        for p in set(modeltext, input):
            resultSet[str(p)] = resultSet.get(str(p), 0) + 1
        inputCombiNum += 1

for g in resultSet:
    if resultSet[g] == inputCombiNum:
        print(g)
```

```
print(time.time() - start_time)
```
