# Supplementary material for: Global Stabilization of Boolean Networks to Control the Heterogeneity of Cellular Responses
Source: Front Physiol. 2018 Jul 17;9:774. doi: 10.3389/fphys.2018.00774 (PMC6060448; doi:10.3389/fphys.2018.00774)
Supplement: Supplementary Dataset S5 — A Python script that conducts global stabilization by Algorithm 1 for the MAPK signaling network (section 4.2). It can be also downloaded from https://github.com/choonlog/Global-stabilization/tree/master/GS_Adjacency_Matrix. [file Data_Sheet_5.pdf]

```
"""
GlobalStablization
Author : Jung-Min Yang(Kyungpook National University), Chun-Kyung Lee(Kyungpook National University), and Kwang-Hyun Cho(Korea Advanced Institute of Science and Technology)
"""
import itertools
import re
import operator
import time

start_time = time.time()
state = {}
nodeUpdate = {}
input = {}
resultSet = {}
inputCombiNum = 0

def canal(lineText):

    lineTextSplit = lineText.split("=")
    leftNode = lineTextSplit[0].strip()
    address = lineTextSplit[1].strip()
    lineText = lineText.replace('(', '').replace(')', '').replace('=', '')
    fullNode = []
    node = []
    duple = []
    nodeDic = {}
    u = 0

    if state[leftNode] == "True" or state[leftNode] == "False":
        return False
    else:
        for x in lineText.split():
            if(x not in ['or', 'and', 'not']):
                fullNode.append(x)

        for j in range(1, len(fullNode)):

            if state[fullNode[j]] == "True":
                nodeDic[fullNode[j]] = "True"
            elif state[fullNode[j]] == "False":
                nodeDic[fullNode[j]] = "False"
            else:
                node.append(fullNode[j])

        combinationNumber = len(node);
        for subset1 in itertools.combinations(node, combinationNumber):
            for k in range(0, combinationNumber + 1):
                for subset2 in itertools.combinations(subset1, k):
                    nodeOnList = [i for i in subset2]
                    nodeOffList = list(subset1)
                    for j in nodeOnList:
```

```
                        nodeOffList.remove(j)
                    for i in nodeOnList:
                        nodeDic[i] = "True"
                    for i in nodeOffList:
                        nodeDic[i] = "False"

                    result = eval(re.sub(r"₩b(₩S+)₩b", lambda m: nodeDic.get(m.group(1), m.group(1)), address))
                    duple.append(result)
                    if u > 0:
                        if duple[0] != duple[u]:
                            break;
                    u += 1

        removedDuple = [duple[i] for i in range(len(duple)) if i == duple.index(duple[i])]

        if len(removedDuple) == 1:
            nodeUpdate[leftNode] = removedDuple[0]

def iteration(modeltext):
    global nodeUpdate
    global state
    step = True

    while step:
        modeltextLine = modeltext.splitlines()
        for m in modeltextLine:
            if not (m == ""):
                canal(m)

        if len(nodeUpdate) > 0:
            for v in nodeUpdate:
                state[v] = str(nodeUpdate[v])
            nodeUpdate = {}
        else:
            step = False

    return(state)
    state = {}

def set(modeltext, input):

    for w in input:
        state[w] = input[w]

    left = {}
    leftOrder = []
    logic = []
    all = []

    modeltextLine = modeltext.splitlines()
    for m in modeltextLine:
        if not (m == ""):
```

```
                lineTextSplit = m.split("=")
                leftOrder.append(lineTextSplit[0].strip())
                left[lineTextSplit[0].strip()] = 0
                logic.append(lineTextSplit[1].strip())

    for j in left:
        for i in logic:
            if i.count(j) > 0:
                left[j] += 1
            else:
                left[j] += 0

    sorted_x = sorted(left.items(), key=operator.itemgetter(1), reverse=True)
    rankLeft = []

    for x in sorted_x:
        rankLeft.append(x[0])
    for g in input:
        rankLeft.remove(g)
        leftOrder.remove(g)

    del rankLeft[5:]

    e = 1
    while e < 3:
        combinationNumber = e;
        for subset1 in itertools.combinations(rankLeft, combinationNumber):
            for k in range(0, combinationNumber + 1):
                for subset2 in itertools.combinations(subset1, k):
                    leftOffList = [i for i in subset2]
                    leftOnList = list(subset1)
                    for j in leftOffList:
                        leftOnList.remove(j)
                    for q in leftOrder:
                        if q in leftOnList:
                            state[q] = "True"
                        elif q in leftOffList:
                            state[q] = "False"
                        else:
                            state[q] = ""

                    result = iteration(modeltext)
                    z = 0
                    for l in result:
                        if result[l]:
                            z += 1
                    if len(left) == z:
                        all.append({"On": leftOnList, "Off": leftOffList})
        e += 1
    return (all)

modeltext = '''
AKT = PDK1 and not PTEN
```

AP1 = JUN and (FOS or ATF2)
Apoptosis = not BCL2 and not ERK and FOXO3 and p53
ATF2 = JNK or p38
ATM = DNA_damage
BCL2 = CREB and AKT
CREB = MSK
DNA_damage = DNA_damage
DUSP1 = CREB
EGFR = (EGFR_stimulus or SPRY) and not (PKC or GRB2)
EGFR_stimulus = EGFR_stimulus
ELK1 = ERK or JNK or p38
ERK = MEK1_2
FGFR3 = FGFR3_stimulus and not (GRB2 or PKC)
FGFR3_stimulus = FGFR3_stimulus
FOS = ERK and RSK and (ELK1 or CREB)
FOXO3 = JNK and not AKT
FRS2 = FGFR3 and not SPRY and not GRB2
GAB1 = GRB2 or PI3K
GADD45 = SMAD or p53
GRB2 = EGFR or FRS2 or TGFBR
Growth_Arrest = p21
JNK = (TAOK and MAP3K1_3) or (MAP3K1_3 and MTK1) or (TAOK and MTK1) or (TAK1 and MTK1) or (TAK1 and MAP3K1_3) or (TAK1 and TAOK) or ((TAOK or MTK1 or MAP3K1_3 or TAK1) and not DUSP1)
JUN = JNK
MAP3K1_3 = RAS
MAX = p38
MDM2 = (p53 or AKT) and not p14
MEK1_2 = (RAF or MAP3K1_3) and not (PPP2CA or AP1)
MSK = ERK or p38
MTK1 = GADD45
MYC = (MSK and MAX) or (MSK and AKT)
p14 = MYC
p21 = not AKT and p53
p38 = (TAOK and MAP3K1_3) or (MAP3K1_3 and MTK1) or (TAOK and MTK1) or (TAK1 and MTK1) or (TAK1 and MAP3K1_3) or (TAK1 and TAOK) or ((TAOK or MTK1 or MAP3K1_3 or TAK1) and not DUSP1)
p53 = (ATM and p38) or ((ATM or p38) and not MDM2)
p70 = PDK1 and ERK
PDK1 = PI3K
PI3K = GAB1 or (RAS and SOS)
PKC = PLCG
PLCG = EGFR or FGFR3
PPP2CA = p38
Proliferation = p70 and MYC and not p21
PTEN = p53
RAF = (RAS or PKC) and not (ERK or AKT)
RAS = SOS or PLCG
RSK = ERK
SMAD = TGFBR
SOS = GRB2 and not RSK
SPRY = ERK
TAK1 = TGFBR
TAOK = ATM
TGFBR = TGFBR_stimulus

```
TGFBR_stimulus = TGFBR_stimulus
'''
inputSet = ["DNA_damage", "EGFR_stimulus", "FGFR3_stimulus", "TGFBR_stimulus"]

for f in range(0, len(inputSet) + 1):
    for subset in itertools.combinations(inputSet, f):
        nodeOnList = [d for d in subset]
        nodeOffList = list(inputSet)
        for t in nodeOnList:
            nodeOffList.remove(t)
        for y in nodeOnList:
            input[y] = "True"
        for y in nodeOffList:
            input[y] = "False"
        for p in set(modeltext, input):
            resultSet[str(p)] = resultSet.get(str(p), 0) + 1
        inputCombiNum += 1

for g in resultSet:
    if resultSet[g] == inputCombiNum:
        print(g)

print(time.time() - start_time)
```
